# Solvent Engineering for Nonpolar Substrate Glycosylation Catalyzed by the UDP-Glucose-Dependent Glycosyltransferase UGT71E5: Intensification of the Synthesis of 15-Hydroxy Cinmethylin β-d-Glucoside

**DOI:** 10.1021/acs.jafc.3c04027

**Published:** 2023-09-01

**Authors:** Jihye Jung, Hui Liu, Annika J. E. Borg, Bernd Nidetzky

**Affiliations:** † Institute of Biotechnology and Biochemical Engineering, Graz University of Technology, NAWI Graz, A-8010 Graz, Austria; ‡ Austrian Centre of Industrial Biotechnology, A-8010 Graz, Austria

**Keywords:** Leloir glycosyltransferase, xenobiotic metabolite, glycosylation, solubility, organic–aqueous biphasic reaction, inclusion complexation, cyclodextrin

## Abstract

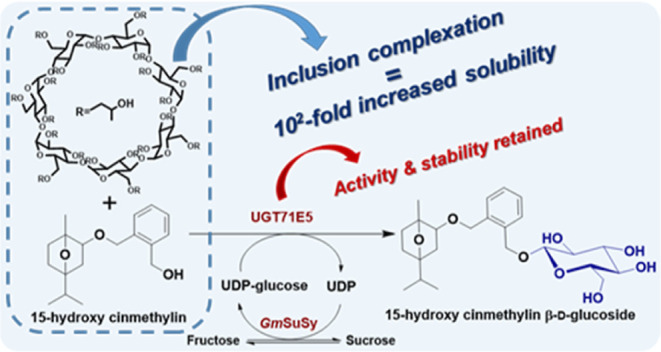

Sugar nucleotide-dependent glycosyltransferases are powerful catalysts of the glycosylation of natural products and xenobiotics. The low solubility of the aglycone substrate often limits the synthetic efficiency of the transformation catalyzed. Here, we explored different approaches of solvent engineering for reaction intensification of β-glycosylation of 15HCM (a C15-hydroxylated, plant detoxification metabolite of the herbicide cinmethylin) catalyzed by safflower UGT71E5 using UDP-glucose as the donor substrate. Use of a cosolvent (DMSO, ethanol, and acetonitrile; ≤50 vol %) or a water-immiscible solvent (*n*-dodecane, *n*-heptane, *n*-hexane, and 1-hexene) was ineffective due to enzyme activity and stability, both impaired ≥10-fold compared to a pure aqueous solvent. Complexation in 2-hydroxypropyl-β-cyclodextrin enabled dissolution of 50 mM 15HCM while retaining the UGT71E5 activity (∼0.32 U/mg) and stability. Using UDP-glucose recycling, 15HCM was converted completely, and 15HCM β-d-glucoside was isolated in 90% yield (∼150 mg). Collectively, this study highlights the requirement for a mild, enzyme-compatible strategy for aglycone solubility enhancement in glycosyltransferase catalysis applied to glycoside synthesis.

## Introduction

Biocatalysis applications in chemical synthesis are often challenged by the low water solubility of the substrates used.^1–6^ With the exception of organic–aqueous interfacial reactions notwithstanding,^1,7^ the enzymatic transformations generally happen in the bulk water phase.^2^ Whenever nonpolar substrates are involved, the solubility enhancement thus becomes a requirement so that the reaction efficiency meets the demands of a practical synthesis.^2,3^ Considering the fundamental importance and applied significance a generally applicable method would entail, numerous strategies of substrate solubilization have been proposed.^1,2,8,9^ These strategies can be broadly categorized according to whether they achieve solubilization in a single aqueous phase or introduce an additional, water-immiscible liquid or solid phase that solubilizes the substrate better than water does.^1,2,10^ There exist countless varieties of either type of the strategy. However, a common pitfall to nearly all approaches is that the enzyme activity, stability, or both are impaired, often severely so, under the conditions used for enhanced solubilization.^11–14^ The challenge, therefore, is to find a strategy that combines solubilization with the retention of the enzyme function. Solvent engineering therefore plays a key role in applied biocatalysis development.^1,2^ While general guidelines for enzyme usage in nonconventional media have emerged from the aggregate evidence of a large set of diverse studies,^1,2,8^ the concrete strategy of substrate solubilization always necessitates a case-specific development under method adaptation to the requirements of the particular enzyme(s) used.^15,16^ Enzyme robustness may not be uniform to the various stressors that occur in the biocatalytic process. However, a generally stable enzyme (e.g., temperature and pH) is promising to also show useful resistance to the solvent conditions applied for substrate solubility enhancement.^1–3,14,15^

Sugar nucleotide-dependent (Leloir) glycosyltransferases (GTs) catalyze the glycosylation of acceptor substrates that represent a broad diversity of chemical structures.^17–19^ Certain classes of GT acceptor, including natural products and xenobiotics in particular, involve a highly nonpolar aglycone core.^17,20–24^ Due to the specificity and flexibility in catalysis offered in useful combination, GTs are promising enzymes for the synthesis of the corresponding acceptor glycosides.^17,22,25–27^ The glycosides involve considerable interest for the diverse uses they have, ranging from chemical reference for studies of the biological metabolism^28,29^ to functional ingredients in food^23,26,30,31^ and cosmetic applications.^23,30,32,33^ However, GTs are generally perceived as “difficult” enzymes for use in applied biocatalysis. Besides their requirement of a nucleotide-activated sugar donor for activity, they also exhibit comparably low robustness,^17,18^ falling short of widely used “industrial workhorse” enzymes such as hydrolases or nicotinamide coenzyme-dependent dehydrogenases.^34,35^ Low robustness of GTs may have been the reason that solvent engineering strategies have not been widely explored with these enzymes.^36^ Nevertheless, glycosylation reactions with nonpolar acceptors are expected to benefit strongly from enhanced solubilization of the substrate into the aqueous phase containing the enzyme.^17,22–24,27^

**Figure 1:**
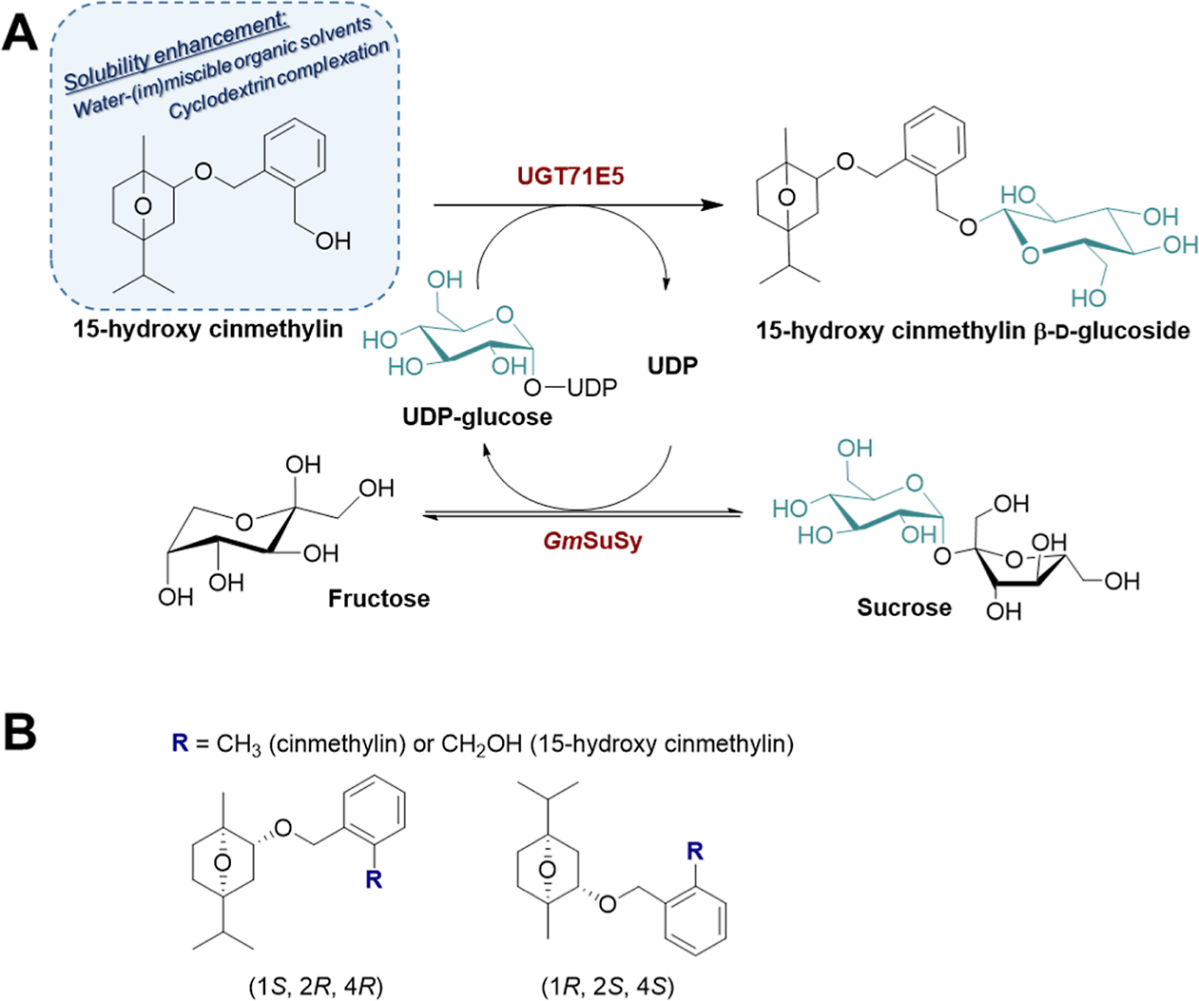
(A) Reaction scheme for the synthesis of 15HCM β-d-glucoside by glycosyltransferase UGT71E5 coupled with UDP-glucose production and regeneration by a sucrose synthase *Gm*SuSy. (B) Chemical structures of cinmethylin/15HCM enantiomers.

In the current study, we focused on β-d-glucosylation of 15-hydroxy cinmethylin (15HCM) from uridine 5′-diphosphate (UDP)-glucose by UGT71E5 of safflower plant (*Carthamus tinctorius*), as shown in Figure 1A.^37,38^ Cinmethylin (Figure 1B) is an agricultural herbicide, and its detoxification in planta involves C15 hydroxylation and glycosylation.^39,40^ 15HCM β-d-glucoside is therefore an important chemical and biological reference of xenobiotic metabolism. UGT71E5 catalyzes the synthesis of 15HCM β-d-glucoside at a useful specific activity (0.43 U/mg) as well as in high selectivity.^37^ The reaction, however, confronts the major challenge of extremely low 15HCM solubility (≤0.1 g/L, in water at 25 °C). To improve the efficiency of 15HCM transformation into 15HCM β-d-glucoside, we here examine the two main strategies of substrate solubilization. We use organic cosolvents (DMSO, ethanol, and acetonitrile) in different concentrations. Additionally, we use water-immiscible organic solvents (*n*-hexane, 1-hexene, *n*-heptane, and *n*-dodecane) and explore different water–organic phase ratios and incubation conditions. Last, we use inclusion complexation^41,42^ in cyclodextrin to enhance solubilization. Earlier works from this laboratory have shown the complexation of phenolic acceptors in 2-hydroxypropyl-β-cyclodextrin (HPβCD) in order to achieve intensification of GT-catalyzed *C*-glycosylation reactions.^36,43–46^ HPβCD was revealed to be well tolerated by different *C*-glycosylating GT enzymes.^36,43,44^ Moreover, HPβCD was readily removed during the product isolation.^36,46^ We find here that organic (co)solvents were not very compatible with UGT71E5 activity and that enzyme stability was also low under the alternative solvent conditions. Applied as an inclusion complex with 2-hydroxypropyl-β-cyclodextrin (HPβCD), 15HCM was dissolved to ∼100 mM in a single aqueous phase, and the UGT71E5 activity was largely unimpaired by the conditions used. Full conversion of 15HCM (50 mM) was shown, and 15HCM β-d-glucoside was recovered in 90% yield (∼150 mg scale). Compared to a reference reaction done with 10 mM 15HCM in 10 vol % DMSO,^37^ the HPβCD-based strategy of solubility enhancement achieved ∼5-fold intensification of the synthetic transformation. In summary, therefore, our study highlights the requirement for a mild, enzyme-compatible strategy of solubilization of the acceptor substrate for the efficient application of GT catalysis to glycoside synthesis.

## Materials and Methods

### Chemicals and Reagents

UDP disodium salt (>97%), UDP-glucose disodium salt (>98%), and HPβCD (>98%, degree of substitution 2–9, product code OH05393, batch number OH053931501) were from Biosynth (Berkshire, United Kingdom). 15HCM (≥99.5%) and 15HCM β-d-glucoside (≥99%) were provided by BASF (Ludwigshafen, Germany). Note: 15HCM is a racemic mixture of the (1*S*, 2*R*, and 4*R*) and (1*R*, 2*S*, and 4*S*) forms (Figure 1B).^37,38^ 15HCM and its glycosylated derivatives are toxic and irritants, and proper care was taken in their handling. All other chemicals and reagents were of the highest available purity and purchased from Carl Roth (Karlsruhe, Germany) or Sigma-Aldrich (Vienna, Austria).

### Enzyme Production

UGT71E5 from *C. tinctorius* (GenBank accession number: KX610759.1) was produced and purified with a slightly modified protocol from that described in the literature.^47^ The codon-optimized synthetic gene of UGT71E5 (Supporting Information) in a pET28a(+) expression vector was expressed in *E. coli* BL21(DE3) cells using Terrific Broth (TB) medium. The enzyme was purified by utilizing its N-terminal 6xHis-tag. Full details of the expression and purification conditions are given in the Supporting Information. Sucrose synthase from soybean (*Glycine max*; *Gm*SuSy, GenBank accession number: AF030231.1) equipped with an N-terminal Strep-Tag II was produced and purified as described in an earlier work.^36,48^ The size and purity of UGT71E5 and *Gm*SuSy were confirmed by SDS-PAGE (Figure S1). Protein concentration was determined based on the absorption at 280 nm on a spectrophotometer (NanoDrop One, Biozym, Vienna, Austria) using a molar extinction coefficient (*Gm*SuSy, 104,210 M^–1^ cm^–1^; UGT71E5, 45,170 M^–1^ cm^–1^) and molecular mass (*Gm*SuSy, 92,244 Da; UGT71E5, 55,167 Da) calculated from the amino acid sequence.

### Enzyme Activity Assays

The specific activity of UGT71E5 toward free and HPβCD-encapsulated 15HCM was determined from a reaction containing 1.0 mM 15HCM and 2.0 mM UDP-glucose.^37^ The activity of *Gm*SuSy on UDP (2.0 mM) was determined from a reaction with 500 mM sucrose. Both activity assays were carried out in Tris buffer (50 mM, pH 7.4) supplemented with 5 mM MgCl_2_. Full details of the activity assays are provided in the Supporting Information. The results are shown in Figures S2 and S3.

### Glycosylation of 15HCM Using Water-(Im)miscible Organic Solvents

#### Reactions with Water-Miscible Organic Solvents

The reactions (0.4–0.5 mL) with DMSO (15–50% v/v) contained 15HCM (20–30 mM), UDP-glucose (20–60 mM), and UGT71E5 (1.5 mg/mL). The reactions with ethanol and acetonitrile (15% v/v) contained 15HCM (20 mM), UDP-glucose (20 mM), and UGT71E5 (1.5 mg/mL) in a final volume of 0.4 mL. All the reactions were performed at 30 °C with agitation (400 rpm, Thermomixer Comfort, Eppendorf, Hamburg, Germany) in Tris buffer (50 mM, pH 9.0) containing 5 mM MgCl_2_ and sampled as described in the Supporting Information under “Enzyme Activity Assays”. The results are shown in Table S1 and Figure S4.

#### Reactions with Substrate Feeding

**Figure 2:**
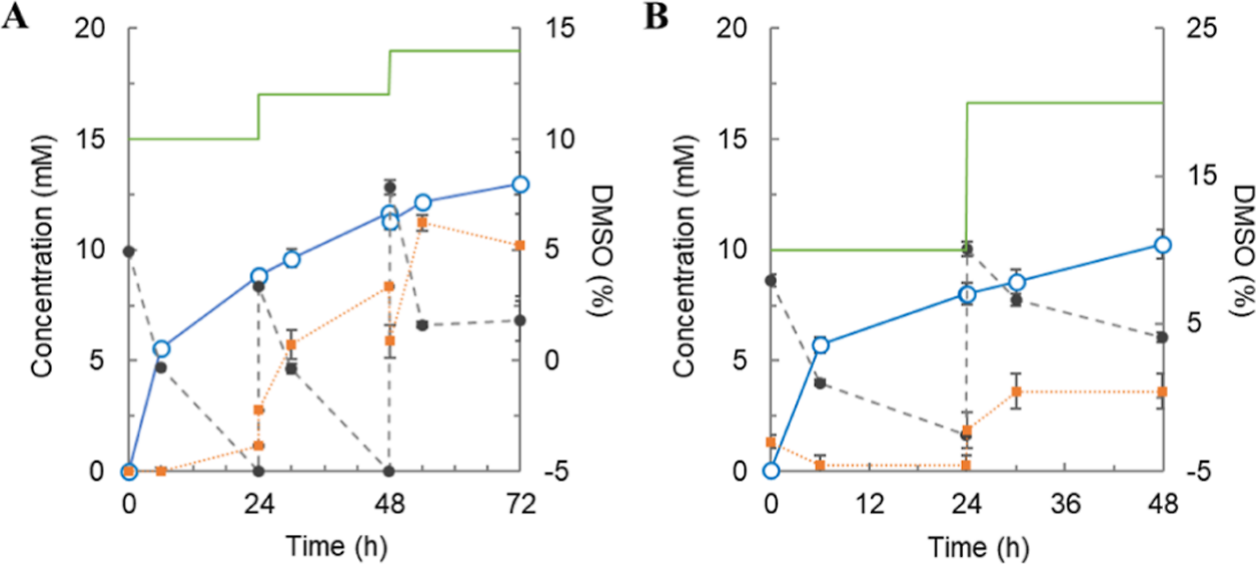
Supplement of 15HCM in its glycosylation by UGT71E5. 15HCM was supplemented with DMSO [green lines: (A) 2% v/v and (B) 10% v/v]. Conversion of 15HCM (closed black circles) to 15HCM β-d-glucoside (open blue circles) was monitored for 72 (A) and 48 h (B). Precipitated 15HCM (orange squares) decreased with 10% DMSO [(B) ∼3.6 mM)], compared to the precipitants with 2% DMSO [(A) ∼11 mM)]. The standard deviations shown are from the analytical determination by HPLC (*n* ≥ 2). The number *N* of experiments was 1.

The experiments with 15HCM feeding included UDP-glucose regeneration by *Gm*SuSy. The reaction mixture at start consisted of 10 mM 15HCM (10% v/v DMSO), UDP (1.0 mM), sucrose (100 mM), and *Gm*SuSy (0.1 mg/mL, 0.41 U) in sodium phosphate buffer (50 mM, pH 7.0) containing MgCl_2_ (5 mM). Note: pH 7.0 was used for the coupled reaction of UGT71E5 and *Gm*SuSy, while pH 9.0 was used for the single-enzyme reaction of UGT71E5. The reason is that high pH favors the equilibrium of the 15HCM glycosylation from UDP-glucose.^37,38^ The reaction was started by the addition of UGT71E5 (0.5 mg/mL, 0.2 U, preincubated at 30 °C for 2 min) and performed at 30 °C under agitation (400 rpm, Thermomixer Comfort). The acceptor substrate was further supplemented at 24 h (10 mM; 2 or 10% v/v DMSO) and 48 h (10 mM; 2% v/v DMSO). The results are shown in Figure 2.

#### Reactions with Water-Immiscible Organic Solvents

**Figure 3:**
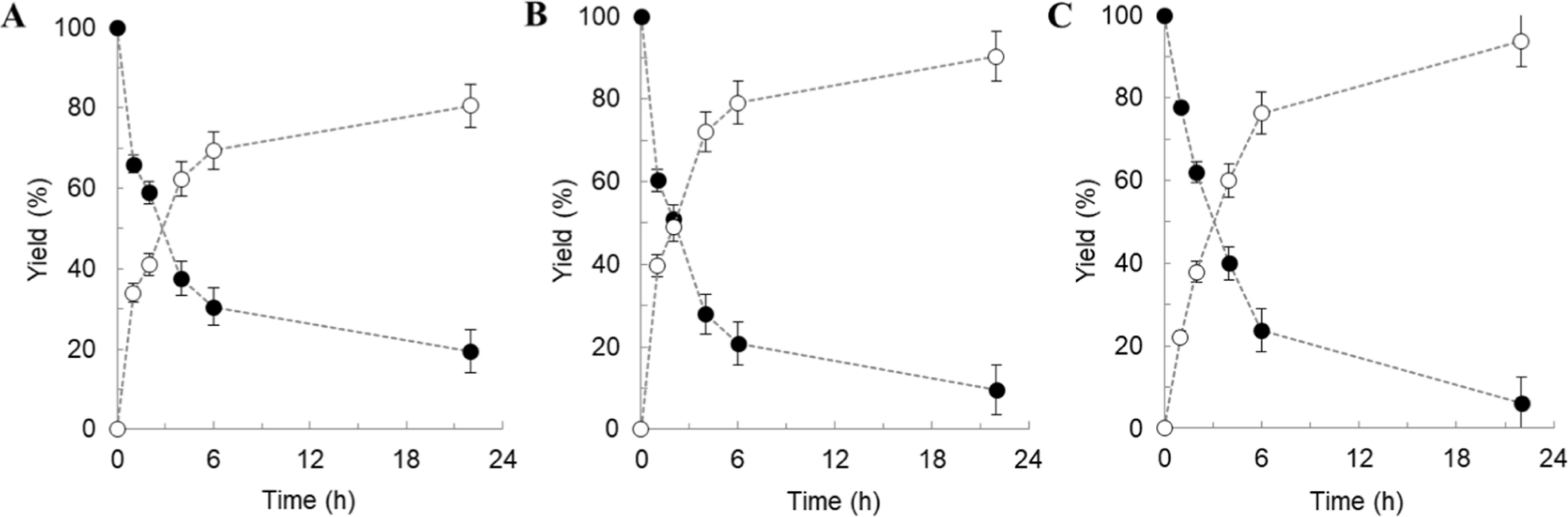
Glycosylation of 15HCM in a 1:4 ratio of organic solvent to aqueous solution in the biphasic system. 15HCM (20 mM: closed circles) was dissolved in organic solvents [0.1 mL: (A) *n*-dodecane, (B) *n*-heptane, and (C) 1-hexene]. UGT71E5 (1 mg/mL) and UDP-glucose (30 mM) were dissolved in an aqueous solution (0.4 mL). 15HCM β-d-glucoside (open circles) was formed and collected in the aqueous phase. The data are summarized in Table 1. The standard deviations shown are from the analytical determination by HPLC (*n* ≥ 2). The number *N* of experiments was 1.

**Figure 4:**
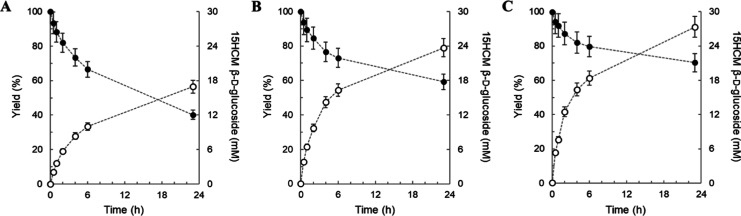
Glycosylation of 15HCM in the organic–aqueous biphasic system with *n*-dodecane and 10% DMSO. 15HCM (30 mM) in *n*-dodecane [(A) 0.3, (B) 0.6, and (C) 0.9 mL] was used. UGT71E5 [(A) 1.5, (B) 3.0, and (C) 4.5 mg/mL)] and UDP-glucose [(A) 60, (B) 120, and (C) 180 mM)] were in the aqueous phase (0.3 mL). DMSO (10% v/v) was added into the aqueous phase. 15HCM (closed circles) was observed in both organic and aqueous phases. 15HCM β-d-glucoside (open circles) was shown in the aqueous phase only. The data are also shown and summarized in Figure S8 and Table 1. The standard deviations shown are from the analytical determination by HPLC (*n* ≥ 2). The number *N* of experiments was 1.

The reactions with a 4:1 ratio of aqueous (0.4 mL) and organic (0.1 mL; *n*-dodecane, *n*-heptane, and 1-hexene) phase contained 15HCM (20 or 100 mM), UDP-glucose (30–250 mM), and UGT71E5 (1.0 mg/mL). Varying agitation rates (300–800 rpm) were used. The experiments with 1:1 ratio of aqueous (0.4 mL) and organic (0.4 mL; *n*-dodecane, *n*-heptane, 1-hexene, and *n*-hexane) phases were performed with 15HCM (30 or 75 mM), UDP-glucose (60 or 70 mM), and UGT71E5 (1.5 mg/mL) under 300 rpm agitation. For the dodecane reactions with additional DMSO (10% v/v of aqueous phase volume, added into the aqueous phase), the ratio of aqueous (0.3 mL) and organic (0.3–0.9 mL) phases was varied from 1:1 to 1:3. The dodecane with additional DMSO reactions was carried out with 15HCM (30 mM), UDP-glucose (60–180 mM), and UGT71E5 (1.5–4.5 mg/mL) under 400 rpm agitation. All the reactions were initiated by the addition of the organic phase (containing 15HCM) into the aqueous phase (containing UDP-glucose and UGT71E5 in 50 mM Tris buffer and 5 mM MgCl_2_, pH 9.0). The reactions were performed at 30 °C (Thermomixer) and sampled as described in the Supporting Information under “Enzyme Activity Assays”. The results are shown in Table 1 and Figures 3, 4, and S5–S8.

**Table 1:** Glycosylation of 15HCM in the Organic–Aqueous Biphasic Reaction with Water-Immiscible Organic Solvents

ratio of organic phase to aqueous phase^a^	water-immiscible organic solvent	[15HCM] in the organic phase (mM)	initial activity (mU/mg)^b^	theoretical [15HCM β-d-glucoside] in the aqueous phase (mM)	observed [15HCM β-d-glucoside] in the aqueous phase after 23 h (mM)^c^	yield in the aqueous phase (%)^d^
1:4	*n*-dodecane	20	28 ± 2	5	4.0 ± 0.3	81
		100^e^	67 ± 4	25	15.1 ± 1.0	61
	*n*-heptane	20	33 ± 2	5	4.5 ± 0.3	90
		100^e^	62 ± 4	25	17.2 ± 1.2	69
	1-hexene	20	18 ± 1	5	4.7 ± 0.3	94
		100^e^	70 ± 5	25	15.9 ± 1.1	64
1:1	*n*-dodecane	30	28 ± 2	30	11.8 ± 0.8	39
		75^f^	70 ± 5	75	20.6 ± 1.4	27
	*n*-heptane	30	30 ± 2	30	12.7 ± 0.9	40
		75^f^	57 ± 4	75	21.0 ± 1.4	28
	1-hexene	30	18 ± 1	30	11.2 ± 0.7^g^	37^g^
		75^f^	37 ± 1	75	20.8 ± 0.1	28
	*n*-hexane	30	42 ± 3	30	16.6 ± 1.1^g^	55^g^
		75^f^	55 ± 4	75	24.6 ± 1.6^g^	33^g^
1:1	*n*-dodecane with 10% DMSO	30	41 ± 3	30	16.9 ± 1.1	57
2:1		30	36 ± 2	60	23.7 ± 1.6	40
3:1		30	28 ± 2	90	27.3 ± 1.8	30

a Volumetric percentage of solvent in total volume.

b Standard deviations are from *N* ≥ 2 determinations in the initial phase (≤6 h) of the reaction.

c Standard deviations are from *n* ≥ 2 analytical determinations.

d .

e Organic phase was 0.1 mL. Aqueous phase was 0.4 mL, containing 250 mM UDP-glucose and 1.0 mg/mL UGT71E5.

f Organic and aqueous phases were each 0.4 mL. In the aqueous phase, UDP-glucose was 70 mM and UGT71E5 was 1.5 mg/mL.

g Values were obtained from both aqueous and organic phases as 15HCM β-d-glucoside was observed in the organic phase.

### Encapsulation of 15HCM by HPβCD

HPβCD inclusion complexes of 15HCM were prepared with a protocol adapted from the literature.^36^ The host–guest molar ratio was varied between 1:1 and 4:1. For the complexation at 3:1 ratio (used for preparing the substrate for cascade reactions), HPβCD (420 mg, 0.30 mmol) was dissolved in deionized water (0.5 mL) by microwave heating (Micro-Chef V98, Moulinex, Austria; 20 s at 750 W) and mixed by inversion every 15 min. For inclusion complex formation, 15HCM (29.04 mg, 0.10 mmol) was added and dissolved as described for HPβCD. The final volume was set to 1.0 mL with deionized water, and the mixture was equilibrated at 70 °C for 30 min. The mixture was further incubated at 22 °C for 3 h and centrifuged for 5 min (21,130 *g*, 22 °C). The concentration of complexed 15HCM was measured based on a calibration curve of free 15HCM on high-performance liquid chromatography (HPLC) (Figures S9 and S10).

### Cascade Reactions of UGT71E5 and *Gm*SuSy on HPβCD-Encapsulated 15HCM

**Figure 5:**
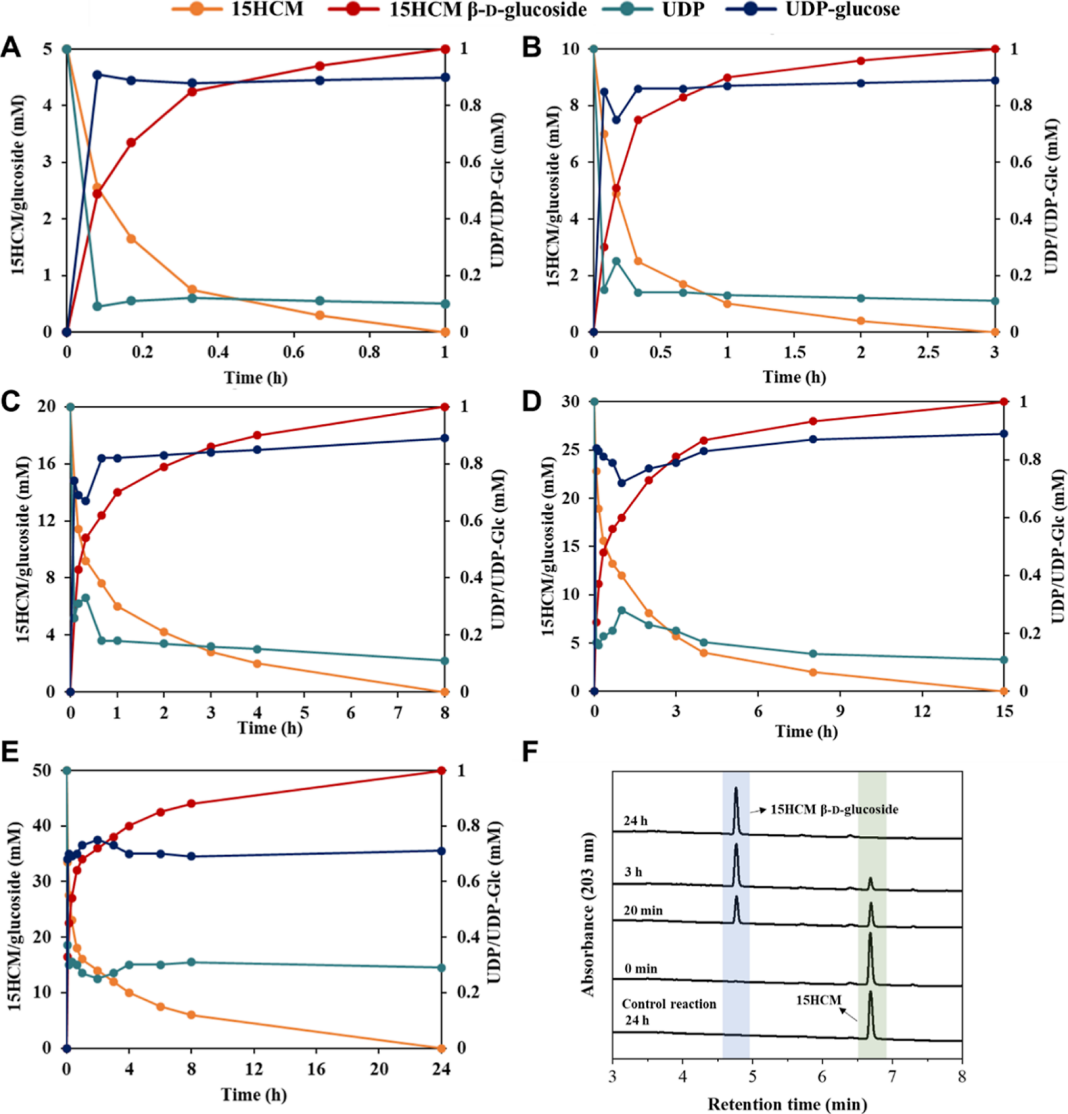
Glycosylation of 15HCM (offered as the HPβCD inclusion complex) by UGT71E5. (A–E) Conversion of 15HCM [orange circles: (A) 5, (B) 10, (C) 20, (D) 30, and (E) 50 mM] to its β-d-glucoside (red circles) conducted at 30 °C in Tris buffer (50 mM, pH 7.4) containing 5 mM MgCl_2_. UGT71E5 was used at following concentrations: (A) 1.2, (B) 1.8, (C) 1.9, (D) 2.4, and (E) 4.8 mg/mL. In panel (E), 3.7 mg/mL UGT71E5 were supplied at the start and 1.1 mg/mL after 8 h of reaction. UDP-glucose (dark blue circles) was regenerated from the reaction of *Gm*SuSy [(A) 0.25, (B) 0.43, (C) 0.43, (D) 0.54, and (E) 2.1 mg/mL] with sucrose (500 mM) and UDP (1 mM, dark cyan circles). (F) HPLC analysis of 50 mM reaction shown in panel (E), including a control reaction performed in the absence of the enzymes (at 24 h time point). The enzyme activities used in the reactions can be calculated with specific activities of 0.32 U/mg for UGT71E5 and 4.1 U/mg for *Gm*SuSy. Data are means of *N* = 2 experiments. The standard deviation was ≤6%.

The reactions were performed in Tris buffer (50 mM, pH 7.4) containing MgCl_2_ (5 mM) in the final volume of 0.3 mL. Sucrose (500 mM), 15HCM-HPβCD complex (5–50 mM), and UDP (1.0 mM) were dissolved in the reaction buffer, followed by the addition of UGT71E5 (1.2–3.7 mg/mL). The reactions were started with *Gm*SuSy (0.25–2.1 mg/mL) addition. In the 50 mM 15HCM-HPβCD reaction with batch addition of the enzymes, 3.7 mg/mL UGT71E5 and 2.1 mg/mL *Gm*SuSy were supplied at the start and 1.1 mg/mL UGT71E5 after 8 h of reaction. The reactions were performed at 30 °C with agitation (500 rpm, Thermomixer) and sampled as described in the Supporting Information under “Enzyme Activity Assays”. Control reactions in the absence of UGT71E5 and *Gm*SuSy were treated identically with the reactions in the presence of the enzymes. The results are listed in Figure 5.

### Production and Isolation of 15HCM β-d-Glucoside

The reactions (24 × 0.3 mL, 7.2 mL total volume) were carried out under the following conditions: 50 mM 15HCM (in HPβCD complex), 500 mM sucrose, and 1.0 mM UDP in 50 mM Tris buffer (pH 7.4, containing 5 mM MgCl_2_) at 30 °C with agitation (500 rpm). UGT71E5 (2.5 mg/mL) and *Gm*SuSy (0.6 mg/mL) were added at start and further supplied after 8 h of reaction (1.6 mg/mL UGT71E5 and 0.4 mg/mL *Gm*SuSy). The reaction was sampled at desired time points by quenching 10 μL of reaction mixture with 90 μL of acetonitrile, and the samples were subjected to thin-layer chromatography (TLC) and HPLC analysis. After reaching full conversion to the glycoside product (after 23 h, Figure S11), the reaction mixtures were pooled, and the enzymes were removed by ultrafiltration (Vivaspin 10 kDa cutoff, Sartorius, Göttingen, Germany). The enzyme-free solution was lyophilized (Christ Alpha 1–4 lyophilizer, B. Braun Biotech International, Melsungen, Germany) and redissolved in ∼4 mL of deionized water. The product mixture was loaded into a column containing silica gel C18 (45 g, 0.035–0.070 mm, Carl-Roth), and deionized water (270 mL) was used for washing out impurities (fructose and sucrose). The target product was eluted with 270 mL of acetonitrile/deionized water (1:1) mixture. The fractions (each 15 mL) were analyzed on TLC and the compounds visualized by UV (15HCM β-d-glucoside) and staining solution (sucrose, fructose, and HPβCD), as shown in Figure S12 A. Fractions containing 15HCM β-d-glucoside were concentrated under reduced pressure (40 °C; Heidolph Laborota 4000 rotary evaporator equipped with a Vacuubrand PC2001 pump and a CVC2000II controller, Wertheim, Schwabach, Germany), lyophilized, and redissolved in a solvent mixture of 1-butanol/acetic acid/deionized water (2:1:1). The product was loaded into a silica 60 column (30 g, 0.04–0.063 mm, Machery-Nagel, Düren, Germany) for HPβCD removal. The column was washed with a 1-butanol/acetic acid/deionized water (2:1:1) mixture until the target product was eluted in the fractions (10 mL, analysis on TLC, Figure S12B). The product-containing fractions were pooled, and the solvent removed was by rotary evaporation and lyophilization. The obtained solid material was dissolved in 150 mL of methanol (for silica removal), and the supernatant was collected after centrifugation (20 min, 16,100 *g*). The pure product was recovered from methanol after rotary evaporation and lyophilization and analyzed by nuclear magnetic resonance (NMR) (Figures S13–S16).

### Analytical Methods

#### Reversed-Phase HPLC

The acceptor substrate (15HCM) and its β-d-glucoside product were separated with an Agilent 1200 Series HPLC-UV system on a Kinetex EVO C18 column (5 μm, 100 Å, 150 × 4.6 mm; Phenomenex, Aschaffenburg, Germany) using a gradient method (1.0 mL/min) of water and acetonitrile (each containing 0.1% (v/v) formic acid) as the mobile phase.^37,38^ Gradient: 20–75% acetonitrile in 5.5 min, 75% acetonitrile for 2 min, 75–20% acetonitrile in 0.1 min, and 20% acetonitrile for 4.5 min. The acceptor substrate and the glucoside product were detected by UV at a 203 nm wavelength. UDP-glucose and UDP were separated on a Kinetex C18 column (5 μm, 100 Å, 50 × 4.6 mm; Phenomenex) using an isocratic method (2.0 mL/min) with 5% acetonitrile and 95% tetrabutylammonium bromide (TBAB) buffer (40 mM TBAB, 20 mM K_2_HPO_4_/KH_2_PO_4_, pH 5.9) as the mobile phase. UDP-glucose and UDP were detected by UV at a 262 nm wavelength. The amount of 15HCM/15HCM β-d-glucoside/UDP/UDP-glucose in monophasic reaction systems was determined based on the relative integrated peak areas. The substrate and product concentrations in biphasic reactions were determined by referring to the peak areas of authentic standards and based on the general principle of mass balance.

#### Thin-Layer Chromatography

TLC was used for the separation of 15HCM, 15HCM β-d-glucoside, sucrose, fructose, and HPβCD. The samples (5 μL) were analyzed on silica gel 60 F_254_ plates (Merck, Darmstadt, Germany). The development solution consisting of 1-butanol/acetic acid/deionized water (2:1:1) was used for the separation. Visualization was performed with UV light (254 nm) and thymol-staining solution consisting of thymol (0.5% w/v), ethanol (95% v/v), and concentrated H_2_SO_4_ (5% v/v). 15HCM and its β-d-glucoside were detected by UV light (254 nm), and those spots were developed by heating the plate to approximately 70 °C for 2 min using a heat gun (Figures S11 and S12).

#### Nuclear Magnetic Resonance

The acquisitions of 15HCM β-d-glucoside were carried out in DMSO-*d*_6_ (99.8% D) on a Bruker Avance III NMR spectrometer (Bruker, Rheinstetten, Germany); 300 MHz for ^1^H NMR, COSY, and HSQC measurements and 75 MHz for a ^13^C NMR measurement were used. The spectra were analyzed using MestReNova 14.3.1 (Mestrelab Research, Santiago de Compostela, Spain). The obtained data are shown in Figures S13–S16.

## Results and Discussion

Three approaches were pursued with the aim of improving the efficiency of the 15HCM β-d-glucoside synthesis. First, the use of organic cosolvents was investigated. Second, the use of water-immiscible organic solvents was examined. Last, inclusion complexation in HPβCD was studied. The scope of each approach was investigated in detail.

### Organic Cosolvents

The UGT71E5 reaction for 15HCM β-d-glucoside synthesis was previously performed using DMSO cosolvent in the range 4–16 vol %.^37^ The 15HCM concentration was 1.0 or 10 mM with UDP-glucose used in a 1.5-fold molar excess. Conversion into 15HCM β-d-glucoside was ≥90% (9.0 mM; 4.1 g/L). Enzyme activity was decreased by ∼30% when DMSO was increased from 4 vol % (0.43 U/mg) to 16 vol % (Table S1).^37^ Here, DMSO was used at 15, 20, and 50 vol %, and to further increase the acceptor concentration, 15HCM was dissolved at up to 30 mM. Time courses of the UGT71E5 reaction are shown in Figure S4. Activities and conversion parameters are summarized in Table S1. UGT71E5 was inactive at 50 vol % DMSO. The reaction at 20 vol % DMSO gave ∼20.5 mM product (∼9.3 g/L; ∼70% conversion). The UGT71E5 activity (0.27 ± 0.02 U/mg; *N* = 3) was 63% that of the standard activity (Table S1). Reactions at 15 and 20 vol % DMSO slowed down strongly after ∼6 h (Figure S4), despite incomplete conversion of 15HCM (≤53%) and UDP-glucose remaining in excess at this point. The results thus suggest that reaction progress was limited by loss of enzyme activity under the conditions used. We therefore explored additional cosolvents (ethanol and acetonitrile) and used each at 15 vol % to dissolve 20 mM 15HCM. Both cosolvents are generally known to be denaturing to proteins, but the immediate effect on UGT71E5 was impossible to predict and thus required experimental assessment. As shown in Figure S4 and Table S1, UGT71E5 exhibited low activity under these conditions. Overall, therefore, the cosolvent approach encountered a major obstacle due to enzyme instability and thus seemed to be of limited use for reaction intensification. We did not consider more-specialized, water-miscible solvents^1–6^ such as ionic liquids or deep eutectic solvents at this stage, in particular because there was no immediate suggestion from the literature that these solvents would be fundamentally less detrimental to enzyme activity than the classical solvents already examined.

### Substrate Feeding

Given the low cosolvent tolerance of UGT71E5, there remained the possibility to supply the total amount of 15HCM substrate not at once but in smaller portions. 15HCM feeding in the way considered would allow for a lower DMSO cosolvent concentration to be used that might be better compatible with enzyme stability. Here, a batch reaction of 10 mM 15HCM was performed in 10 vol % DMSO (Figure 2), and after 24 h, the same amount of 15HCM (dissolved in pure DMSO) was added to increase the total cosolvent concentration by 2 vol % (Figure 2A) and 10 vol % (Figure 2B). The reactions were tracked by analyzing 15HCM and 15HCM β-d-glucoside in solution, and the insoluble (precipitated) 15HCM was calculated from the mass balance. Figure 2A shows that after the first substrate addition, 15HCM precipitated in large amounts. This was unexpected because the 15HCM concentration was not increased substantially above the one used initially, and the DMSO concentration was increased from 10 to 12 vol %. As shown in Figure 2A, the release of 15HCM β-d-glucoside continued at a slow rate. The 15HCM addition was repeated after 48 h, leading to further precipitation and no increase in the 15HCM consumption rate. Performing the 15HCM addition with increase in total DMSO concentration to 20 vol % (Figure 2B) resulted in a smaller amount of substrate precipitation; yet, the 15HCM β-d-glucoside formation was slow. The product concentrations (≤13 mM) and conversions (≤43%) reached in the experiments were not convincing to justify a relatively complex mode of operating the reaction. Solubility control of 15HCM appeared to be difficult under conditions of accumulating 15HCM β-d-glucoside. Slowdown of the reaction was not prevented, suggesting impaired UGT71E5 activity. Earlier work on the GT-catalyzed natural product *C*-glycosylation^43^ has shown that the enzyme activity and stability can be affected negatively by the insoluble substrate precipitated in the reaction. Considering the goal of this study to enhance the 15HCM β-d-glucoside synthesis by ∼10-fold to a product concentration of ∼50 mM, we realized the requirement of a different approach for 15HCM solubility enhancement.

### Water-Immiscible Organic Solvents

The aqueous–organic two-phase reaction does not strictly enhance the solubility in the aqueous phase; yet, it constitutes a convenient way of enhancing the amount of dissolved substrate introduced into the reaction mixture. Substrate supply into the aqueous phase occurs by liquid–liquid mass transfer that is typically more efficient and more easily controlled than substrate dissolution from a solid precipitate. Following the well-established general guidelines of a water-immiscible solvent use for enzymatic reactions,^11,15,49,50^ we selected strongly hydrophobic solvents characterized by a log *P*^OW^ (octanol–water partition coefficient) of ≥2 (see Table S2 for a parameter summary of the used solvent). There is furthermore evidence that linear nonbranched alkanes are less detrimental to enzymes than esters, cyclic ethers, simple ethers, aromatic chemicals, and cyclic alkanes.^15^ We therefore chose 1-hexene, *n*-hexane, *n*-heptane, and *n*-dodecane covering the log *P*^OW^ range of ∼3 to ∼7 (Table S2). We note the relatively high evaporation rate of all solvents except *n*-dodecane (Table S2), rendering them nonpreferred from the health–environment–safety point of view.^49^ However, for a preliminary solvent screening to assess a two-phase reaction for 15HCM β-d-glucoside synthesis in principle, all solvents were suitable. As discussed later, a possible advantage of the two-phase mixture is selective partitioning of 15HCM and 15HCM β-d-glucoside to the organic and the aqueous phase, respectively.

The first set of experiments was performed at 4:1 ratio of aqueous and organic phases, with agitation at 300 rpm (Thermomixer) and supply of 15HCM solely through the organic solvent at 20 or 100 mM. The enzyme activities determined from the initial reaction rates were ∼28 mU/mg for *n*-dodecane, ∼33 mU/mg for *n*-heptane, and ∼18 mU/mg for 1-hexene. The activities increased up to 3.8-fold in response to the 5-fold increase in 15HCM concentration in the organic solvent (Table 1). Note that variation in the UDP-glucose concentration used (30 or 250 mM; Table 1) did not cause change in the reaction rate. Activities measured in the two-phase reactions were lowered by ≥13-fold compared to the standard activity determined in a single aqueous phase (1.0 mM 15HCM, 4 vol % DMSO, Table S1). The results suggested that the 15HCM concentration in the aqueous phase might have been too low to saturate UGT71E5 for full activity. Time courses of the enzymatic reactions at 20 mM 15HCM in the organic phase are shown in Figure 3. 15HCM β-d-glucoside accumulating in the aqueous phase indicated high conversion (≥81%) of 15HCM supplied. Direct measurements of 15HCM from the organic phase confirmed the results based on the close mass balance. Partial evaporation of *n*-hexane (≥20%) and 1-hexene (≥50%) was, however, noted. The conversion was lower (60–69%) when 15HCM was supplied at 100 mM in the organic phase (Table 1 and Figure S5). The agitation rate was varied in the range of 300–800 rpm, considering that enhanced energy input by agitation could benefit the interfacial mass transfer through its combined effect on the overall mass-transfer coefficient and the specific liquid–liquid exchange surface area. An increase in specific surface area could, however, also result in enhanced enzyme inactivation. Results in Figure S5B show that the UGT71E5 activity was increased (∼1.8-fold) due to enhanced agitation, consistent with the idea that liquid–liquid mass transfer is partially rate-limiting for the overall reaction rate (“activity”) under standard agitation conditions (300 rpm). The final 15HCM β-d-glucoside concentration reached was hardly dependent on the agitation rate (Figure S5). Time courses of reaction (Figure S5C–E) show differences in 15HCM β-d-glucoside release at varied agitation rates, primarily in the early phase of the conversion. We interpret these results to indicate that increased agitation did not accelerate the enzyme inactivation substantially. If this were the case, one would expect the 15HCM conversion to decrease at an increased agitation rate.

Having shown the two-phase UGT71E5 reaction in principle, we increased in the next step the volume portion of the organic solvent to an aqueous–organic phase ratio of 1:1. The 15HCM concentration in the organic solvent was 30 mM and 75 mM. The enzyme activities were slightly increased in *n*-dodecane (70 mU/mg, 75 mM 15HCM) and even decreased up to 1.9-fold in *n*-heptane (57 mU/mg, 75 mM 15HCM) and 1-hexene (37 mU/mg, 75 mM 15HCM) compared to the previous reactions at 4:1 phase ratio, as shown in Table 1, and they still did not reach the benchmark of the standard assay (431 mU/mg). The product yields decreased with increasing 15HCM concentration in the organic phase, from 37–55% at 30 mM to 27–33% at 75 mM. Time courses of the 30 mM reactions (Figure S6) show that the 15HCM conversion into β-d-glucoside slowed down after ∼3 h, and there was little progress of reaction between 6 and 24 h. The kinetic behavior was highly similar for all reactions and appeared to be independent of the organic solvent used. It was comparable to the kinetic behavior seen in the other time courses previously shown in Figures 3 and S5C–E. The point of decline in the reaction rate was always localized at a similar time; yet, it seemed to be unrelated to the degree of conversion reached or the concentration of 15HCM β-d-glucoside released. The plausible explanation of the observed behavior was general instability of UGT71E5 under the conditions used. We also analyzed the distribution of 15HCM and 15HCM β-d-glucoside between the aqueous and organic phase in the different solvent reactions. Results in Figure S7 reveal that except for minor concentrations found in 1-hexene (0.10 ± 0.02 mM; *N* ≥ 3) and *n*-hexane (0.11 ± 0.03 mM; *N* ≥ 3), 15HCM β-d-glucoside was present exclusively in the aqueous phase. 15HCM by contrast was present primarily in the organic phase, with only small concentrations found in the water phase (1.4–1.7 mM; *N* ≥ 3).

Considering the possibility that the two-phase reactions were limited by the 15HCM substrate available in the aqueous phase, we performed further experiments with *n*-dodecane solvent at 30 mM 15HCM and used DMSO (10 vol % of aqueous phase volume) as cosolvent additionally. The idea was to tune the phase partitioning of 15HCM so that the substrate concentration in the water phase was enhanced. The aggregate evidence up to this point suggested that *n*-dodecane was preferred among the series of solvents here explored. The aqueous/organic phase ratio varied from 1:1 to 1:3. Time courses of 15HCM β-d-glucoside release are shown in Figure 4. The UGT71E5 activity did not benefit from the change in the reaction conditions (Table 1). The product concentration in the aqueous phase increased from 17 to 27 mM as the volume portion of *n*-dodecane increased 3-fold. However, the 15HCM β-d-glucoside yield based on the total 15HCM converted decreased from 57 to 30% (Table 1). Analysis of the phase partitioning of 15HCM and 15HCM β-d-glucoside (Figure S8) showed an increase in 15HCM available in the water phase due to the change in the phase ratio by 1.9-fold, from 1.9 mM at 1:1 ratio (panels A and D) to 3.7 mM at 1:3 ratio (panels C and F). 15HCM β-d-glucoside was present in the water phase. The effect of the DMSO cosolvent on the aqueous 15HCM concentration was moderate, and a 36% increase was calculated from the results in Figure S7 (panel E; ∼1.4 mM) and the Supporting Information, Figure S8 (panel D; ∼1.9 mM).

As an intermediate conclusion, the study of the UGT71E5 reaction in an aqueous–organic two-phase mixture provided an important insight into substrate and product phase partitioning and the kinetic behavior of the enzymatic transformations under the different conditions of organic solvent and reaction operation used. At this stage, however, we also realized that it would be difficult to use the two-phase reaction for a 15HCM β-d-glucoside synthesis that combined high product concentration (target: ≥50 mM) with a suitable conversion of the 15HCM substrate (target: ≥90%). Although the range of possible solvents to be examined is still large, with deep eutectic solvents and emerging biobased solvents such as Cyrene representing interesting classes,^1–6^ we were not convinced based on the evidence already shown that the approach of water-immiscible solvent would hold promise in particular. We therefore considered 15HCM solubility enhancement by inclusion complexation in HPβCD as an alternative.

### HPβCD Inclusion Complex

Our earlier studies on GT-catalyzed *C*-glycosylation of flavonoid acceptors have noted the requirement of substrate solubility enhancement for synthetic reaction at high efficiency.^36,43–46^ Unlike here, solvent engineering approaches have not been investigated in detail for *C*-glycosylation. By applying the encapsulation of phloretin (a flavonoid) in HPβCD, enzymatic syntheses of the phloretin 3′-β-d-glucoside (nothofagin)^36,44,45^ and the phloretin 3′,5′-di-β-d-glucoside^46^ were shown at concentrations of up to 50 mM and higher, exceeding the water solubility of the noncomplexed phloretin by about 10^2^-fold. Conversion of the phloretin acceptor was excellent as well (≥90%).^36,44,46^ Solubilization by inclusion complex formation with HPβCD, clearly, increases the complexity of the overall synthetic procedure compared to the use of a standard organic (co)-solvent,^36,43,46^ and it would also add to the production costs of a biocatalytic process. Given the evidence from previous research that (a) HPβCD was well tolerated by different GT enzymes (including the sucrose synthase used here for UDP-glucose regeneration^36,43,44^) and (b) it was removed relatively easily during the product isolation,^36,46^ we considered the strategy of complexation by HPβCD to be promising for reaction intensification in 15HCM β-d-glucoside synthesis.

15HCM complexation was examined at a concentration of 50 mM, and the degree of solubilization was measured in the presence of varied HPβCD concentrations, representing a host–guest molar ratio between 1:1 and 4:1. A 3-fold excess of HPβCD was required to dissolve 15HCM to ≥95% (Figures S9 and S10). Using the host–guest molar ratio of 3:1, 15HCM was successfully encapsulated up to a 100 mM concentration. When offered 15HCM as an inclusion complex with HPβCD, the UGT71E5 activity was retained (0.32 ± 0.02 U/mg; ∼79 ± 6%; *N* = 2) compared to the reaction in the standard assay (Figure S2). Reactions were performed at different 15HCM concentrations in the range of 5.0–50 mM. The UDP-glucose donor substrate was generated in situ from UDP (1.0 mM) by the sucrose synthase reaction from sucrose present in excess (500 mM; *Gm*SuSy activity assay in Figure S3). The results are shown in Figure 5. Besides 15HCM and 15HCM β-d-glucoside, UDP and the intermediary UDP-glucose were also tracked analytically. The conversion of 15HCM into β-d-glucoside proceeded to completion in all reactions (Figure 5A–E). The initial rate of 15HCM β-d-glucoside release corresponded to expectation from the enzyme concentration used and the specific activity of UGT71E5. The results show that the UGT71E5 activity was utilized efficiently in the reactions, enabled by a suitable supply of UDP-glucose from sucrose and UDP. The results also imply that the UDP-glucose formation was never rate-limiting for the overall conversion, supporting the design of the cascade reactions in Figure 5 with *Gm*SuSy activity used in ≥3-fold excess. Measurements of UDP and UDP-glucose affirm the conclusion of rate limitation. In all reactions (Figure 5A–E), the initial UDP concentration (∼1.0 mM) dropped to a much lower value that remained largely constant throughout the conversion. The distribution of total UDP into the steady-state levels of UDP-glucose and UDP implies a UDP-glucose formation rate (*Gm*SuSy) that exceeded the corresponding consumption rate (UGT71E5).

All reactions in Figure 5 feature a decrease in the product release rate at some point of the time course, typically when ∼50% conversion of the available 15HCM was achieved. The decline in rate might arise due to several factors in combination that time course analysis alone cannot resolve. The availability of a free 15HCM substrate from the inclusion complex with HPβCD to UGT71E5 could be an issue. Further work beyond the scope of this research is needed for clarification. However, the experiment with the addition of fresh enzyme after 8 h (Figure 5E) shows that inactivation of UGT71E5 does not control the productivity of the overall reaction of the HPβCD complex of 15HCM. The reaction rate was only modestly enhanced upon the enzyme addition to increase of the total loading of UGT71E5 by 30% (Figure 5E). Difference to enzymatic reactions in the presence of organic (co)solvent is noted for these reactions involve loss of UGT71E5 activity as a main factor of conversion efficiency. The HPLC traces (Figure 5F) display the successful conversion of 50 mM 15HCM into the corresponding β-d-glucoside achieved in 24 h, while the control reaction in the absence of enzymes shows intact 15HCM (Figure 5F). Overall, the UGT71E5-*Gm*SuSy cascade reaction was efficient for 15HCM β-d-glucoside synthesis at substantial intensification of the final product concentration (≥5-fold) when the 15HCM substrate was supplied as an inclusion complex with HPβCD.

### Preparative Biotransformation with Product Isolation

Preparative synthesis was performed at ∼160 mg scale of 15HCM β-d-glucoside. 15HCM was offered as the HPβCD inclusion complex at 50 mM concentration of the acceptor substrate. Conversion of 15HCM to the corresponding β-d-glucoside was monitored by HPLC and TLC, and the enzymes were removed at the point of complete conversion (≥99%, after 23 h; Figure S11). The product was isolated by two-step silica column chromatography. The first step was performed on a silica C18 resin, from which the target product was coeluted with HPβCD (Figure S12A). Secondary chromatography was conducted on a silica 60 column using the TLC eluent as a mobile phase, leading to successful separation of 15HCM β-d-glucoside from HPβCD (Figure S12B). However, operating the column with a mobile phase consisting of 1-butanol, acetic acid, and water partially dissolved silica into the product fractions. Methanol extraction was used for removing the dissolved silica, and the final extract showed over 95% purity of 15HCM β-d-glucoside. The target product was obtained in excellent yield (147 mg, 90%); yet, the explorative purification method could be further streamlined by excluding the C18 column. Identity of the obtained 15HCM β-d-glucoside was confirmed by ^1^H- and ^13^C NMR, HSQC, and COSY analysis (Figures S13–S16).

In summary, we have performed a systematic study of 15HCM solubility enhancement for UGT71E5 reaction intensification, with the aim of making 15HCM β-d-glucoside synthesis more efficient. Strategies of solvent engineering for improved solubilization of hydrophobic substrate have not been assessed broadly for biotransformations by enzymes of the Leloir glycosyltransferase class.^17,18,43–46^ Here, approaches based on organic solvents miscible or immiscible with the aqueous buffer failed due to limited enzyme robustness. Inclusion complexation with HPβCD represented a mild, enzyme-compatible approach to 15HCM solubility enhancement. The study of 15HCM glycosylation by UGT71E5 can be relevant in general for the cascade catalysis of Leloir glycosyltransferases applied to glycoside synthesis.

## Supporting Information Available

Materials and methods; codon-optimized gene sequence of UGT71E5; glycosylation of 15HCM with water-miscible organic solvents; physiochemical properties of organic solvents; purification of UGT71E5 and *Gm*SuSy by affinity chromatography; time courses from the activity assays of UGT71E5 with free and HPβCD-encapsulated 15HCM; time course from the activity assay of *Gm*SuSy; glycosylation of 15HCM with water-miscible organic solvents; acceleration of substrate transfer in the biphasic reaction; glycosylation of 15HCM in a 1:1 organic–aqueous biphasic system; partition of 15HCM β-d-glucoside and 15HCM in a 1:1 organic–aqueous biphasic reaction; calibration line for 15HCM; encapsulation efficiency and UGT71E5 activity with different concentrations of HPβCD; HPLC and TLC analysis from the production of 15HCM β-d-glucoside for product isolation; TLC analysis from the silica column chromatographic isolation of 15HCM β-d-glucoside; and NMR spectra of isolated 15HCM β-d-glucoside (PDF)

## Abbreviations

GTs: glycosyltransferases
15HCM: 15-hydroxy cinmethylin
UDP: uridine 5′-diphosphate
DMSO: dimethyl sulfoxide
HPβCD: 2-hydroxypropyl-β-cyclodextrin
TB: terrific broth
*Gm*SuSy: sucrose synthase from soybean *Glycine max*
SDS-PAGE: sodium dodecyl sulfate polyacrylamide gel electrophoresis
HPLC: high-performance liquid chromatography
TLC: thin-layer chromatography
NMR: nuclear magnetic resonance
TBAB: tetrabutylammonium bromide
COSY: correlation spectroscopy
HSQC: heteronuclear single-quantum coherence
log *P*^OW^: the octanol–water partition coefficient
