# Supplementary material for: Solvent Engineering for Nonpolar Substrate Glycosylation Catalyzed by the UDP-Glucose-Dependent Glycosyltransferase UGT71E5: Intensification of the Synthesis of 15-Hydroxy Cinmethylin β-d-Glucoside
Source: J Agric Food Chem. 2023 Sep 1;71(36):13419–29. doi: 10.1021/acs.jafc.3c04027 (PMC10510383; doi:10.1021/acs.jafc.3c04027)
Supplement: Supplementary file 1 — jf3c04027_si_001.pdf [file jf3c04027_si_001.pdf]

Solvent Engineering for Nonpolar Substrate Glycosylation Catalyzed by the UDP-glucose Dependent Glycosyltransferase UGT71E5: Intensification of the Synthesis of 15-Hydroxy cinmethylin β-D-glucoside

Jihye Jung[a,§], Hui Liu[a], Annika J. E. Borg[a,b], Bernd Nidetzky[a,b,*]

[a] Institute of Biotechnology and Biochemical Engineering, Graz University of Technology, NAWI Graz, A-8010 Graz, Austria

[b] Austrian Centre of Industrial Biotechnology, A-8010 Graz, Austria

[§] Present address: Natural Products Research Institute in College of Pharmacy, Seoul National University, 1 Gwanak-ro, Gwanak-gu, Seoul, 08826, Republic of Korea.

[*] Corresponding author. Phone: +43-316-873-8400; *E-mail: bernd.nidetzky@tugraz.at

Supporting information

Table of contents

Materials and methods – addition to main text

Codon-optimized gene sequence of UGT71E5

Supporting Table S1. Glycosylation of 15HCM with water miscible organic solvents

Supporting Table S2. Physiochemical properties of organic solvents

Supporting Figure S1. Purification of UGT71E5 and *Gm*SuSy by affinity chromatography

Supporting Figure S2. Time courses from the activity assays of UGT71E5 with free and HPβCD-encapsulated 15HCM

Supporting Figure S3. Time course from the activity assay of *Gm*SuSy

Supporting Figure S4. Glycosylation of 15HCM with water miscible organic solvents

Supporting Figure S5. Acceleration of substrate transfer in biphasic reaction

Supporting Figure S6. Glycosylation of 15HCM in 1:1 organic-aqueous biphasic system

Supporting Figure S7. Partition of 15HCM β-D-glucoside and 15HCM in 1:1 organic-aqueous biphasic reaction

Supporting Figure S8. Partition of 15HCM β-D-glucoside and 15HCM in organic-aqueous biphasic reaction with n-dodecane and 10% DMSO

Supporting Figure S9. Calibration line for 15HCM

Supporting Figure S10. Encapsulation efficiency and UGT71E5 activity with different concentrations of HPβCD

Supporting Figure S11. HPLC and TLC analysis from the production of 15HCM β-D-glucoside for product isolation

Supporting Figure S12. TLC analysis from the silica column chromatographic isolation of 15HCM β-D-glucoside

Supporting Figure S13-S16. NMR spectra of isolated 15HCM β-D-glucoside

References

Materials and methods – addition to main text

Enzyme production

The production of UGT71E5 was performed as described in literature[1], with some modifications to the protocol. UGT71E5 was expressed in *E. coli* BL21(DE3) cells (instead of *Transetta*(DE3)) in Terrific Broth (TB) medium (instead of Lysogeny Broth) supplemented with kanamycin (50 µg/mL). The expression was induced with isopropyl β-D-thiogalactoside (IPTG; 0.2 mM) at cell density (OD_{600}) of 0.8 (instead of 0.4-0.6) and the time of gene expression at 18 °C was extended from 12 h to 20 h. The cells were harvested by centrifugation (3,010 × *g*, 4 °C, 20 min) and the pellet resuspended in His-tag binding buffer (20 mM Na_2HPO_4, 150 mM NaCl, 20 mM imidazole, pH 7.4) containing phenylmethylsulfonyl fluoride (PMSF, 1 mM). The cells were disrupted by sonication (Sonic Dismembrator Model 505, Fisher Scientific, Vienna, Austria; pulse 2 sec on, 4 sec off, 30% amplitude, 6 min) and centrifuged (10,000 × *g*) at 4 °C for 60 min. The supernatant was collected and filtered (0.22 µm) prior to loading onto the HisTrap™ FF column (5 ml resin, Cytiva, Chicago, IL). UGT71E5 bearing an N-terminal 6xHis-tag was eluted with a gradient of 60 min using a buffer (20 mM Na_2HPO_4, 150 mM NaCl, pH 7.4) containing 300 mM imidazole. The pure fractions of UGT71E5 were pooled and re-buffered against 50 mM Tris-HCl buffer (pH 7.4) supplemented with 5% (v/v) glycerol using Vivaspin Turbo tubes (10,000 MWCO PES; Sartorius, Goettingen, Germany). After buffer exchange, the protein was divided into aliquots, flash frozen in liquid nitrogen, and stored at -80 °C.

Enzyme activity assays

The activity assay of UGT71E5 (83.3 ng/mL) contained 1.0 mM 15HCM (dissolved in DMSO; 4% final concentration, or HPβCD-complexes) and 2.0 mM UDP-glucose in final volume of 0.3 mL. The activity assays of *Gm*SuSy (7.0 ng/mL) were performed in 0.5 mL volume (containing 2.0 mM UDP and 500 mM sucrose). The reactions (UGT71E5, *Gm*SuSy) were incubated at 30 °C with agitation (500 rpm; Thermomixer Comfort, Eppendorf, Hamburg, Germany) and quenched with ice-cold acetonitrile (50% v/v) final concentration) at desired time points. The precipitated enzyme was removed by centrifugation (21,130 × *g*, 4 °C, 20 min) prior to HPLC analysis. The initial rates were determined from the corresponding linear

parts of the time-courses, by dividing the slope of the linear regression (mM/min) by the enzyme concentration (mg/mL) giving the initial rate in μmol/(min mg protein). One unit (U) of UGT71E5 (or *Gm*SuSy) activity is defined as the amount of enzyme producing 1 μmol of 15HCM-β-D-glucoside (for *Gm*SuSy: UDP-glucose) per minute under conditions used, where the acceptor substrate is present in excess. UGT71E5 and *Gm*SuSy showed activities (0.4 U/mg, UGT71E5 with non-complexed 15HCM; 3.6 U/mg, *Gm*SuSy) consistent with previous reports on these enzymes.[2,3]

Codon-optimized gene sequence of UGT71E5

GGTCCGCATCAGAAAACCATGACCAATAGTGAACTGGTGTTTATTCCGAGTCCGGGCGCAGG
TCATCTGCCGCCTACCGTGGAACTGGCAAAACTGCTGCTGCGCCGTCATCATCGTCTGAGCA
TTACCATTATTATTATGAAAGCACCGTTCGGCGGTGGCGCATATGATACCGCCAAACTGGAT
AGCACCCCGCGCCTGCGCTGTGTGGAAATTCCGAGCGATGATAGCACCGCCGCACTGATTAG
CCCGACCGCATTTCTGACCGCATTCATTGATCATCATAAACCGCATGTGCGTAATATTGTGCG
TGAAAGTATTGCCGATCCGGGCGGCACCGTTCGTCTGGCTGGCTTTGTGGTGGATATGTTTTG
TGTGGATATGGTTGATGTTGCAAATGAATTTGGCGCACCGACCTATGCATATTTTACCAGTG
GCGCCGCAATGCTGGGTCTGATGTTTCATCTGCAGGCAAAACGTGATGATGATAATCTGGAT
GTTACCGAACTGGATAAAAATAGTCGTAGTGTTATTAGCGTGCCGAGCTATATTAATCCGGT
GCCGGTTAATGTTCTGCTGGATGTGCTGTTTGATAAAAATGGTGCAAAAATGTTCCTGGATC
TGGCAAAACGCTTTCGTGAAACCAAAGGTATTATTGTTAACAGCTTTCGTGAACTGGAAAGC
CATGCAATTGAAGTTCTGAGCGATGATCCGGATATTCCGCCGGTGTTTCCGGTTGGCCCGAT
TCTGAATCTGAATAGCACCACCGATGATGGTAAAGTGGATGATATTATGACCTGGCTGGATG
AACAGCCGGAACGCAGTGTTGTTTTTCTGTGTTTTGGCAGCATGGGTACCTTTCCGGAAGAA
CAGATTCGCGAAATTGCAAGTGCAATTGAAAGCGGTGGTCATCGCTTTCTGTGGAGCCTGCG
CCGTCCGAGCAGCAAAGAAAAAATGGAAAGCCCGAAAGAATATGAAGATCCGGGTGAAGT
TCTGCCGGAAGGTTTTCTGGAACGTACCAGTGGCGTTGGTCGTGTGATTGGCTGGGCACCGC
AGCTGATGGTGCTGAGTCATCGCAGTGTTGGCAGTTTTGTTAGTCATTGTGGCTGGAATAGT
ACCCTGGAAAGCATTTGGTGCGGTGTGCCGATTGCCGCATGGCCGATGTATGCCGAACAGCA
GACCAATGCCTTTCAGCTGGTTAGCGAAGCAGGTATTGCCGCCGAAGTTCGTATGGATTATC
GTACCAATATGAAACCGGGCGGTGAACAGATGATTGTTACCGCAGAAGAAATTGAACGTGG
CATTCGCCGTGTGATGAGTGATGGCGAAATTAAGCGCAAAGCAGAAGAAATGAAAGAAAAA
AGTCGTCTGGCAGTTAGCGAAGGTGGCAGTAGCTATGATAGCATTGGTGACTTTATTCATCA
TGTTATGAACGAATAA

Supporting Table S1. Glycosylation of 15HCM with water miscible organic solvents.

water miscible organic solvent	concentration of solvent	15HCM (mM)	initial activity (mU/mg)	15HCM β-D-glucoside (mM)	product yield (%) [a]	reference
DMSO	4%	1	431	0.95	95	[2]
	10%	10	360	8.98	89	[2]
	12%	1	335	> 0.99	99	[2]
	16%	1	305	> 0.99	99	[2]
	15% [b]	20	61 ± 3 [c]	9.4 ± 0.5 [d]	47	this study
	20% [e]	30	265 ± 18 [c]	20.5 ± 1.4 [d]	68	this study
	50% [e]	30	N.D.	N. D.	N.D.	this study
ethanol	15%[b]	20	0.15 ± 0.01 [c]	0.30 ± 0.01 [d]	1.5	this study
acetonitrile	15%[b]	20	N.D.	N. D.	N.D.	this study

[a] Product yield (%) = $\frac{\text{concentration of released 15HCM β-D-glucoside}}{\text{initial concentration of 15HCM}} \times 100$

[b] 15HCM and UDP-glucose were each 20 mM. UGT71E5 was 1.5 mg/mL. Total volume was 0.5 mL.

[c] Standard deviations are from N ≥ 2 determinations in the initial phase (≤ 6 h) of the reaction.

[d] Standard deviations are from n ≥ 2 analytical determinations.

[e] 15HCM was 30 mM. UDP-glucose was 60 mM. UGT71E5 was 1.5 mg/mL. Total volume was 0.4 mL.

N.D. not detected.

Supporting Table S2. Physiochemical properties of organic solvents. [a]

organic solvents	molecular formula	molecular weight (g/mol)	log P^{ow}	relative evaporation rate [b]	water solubility at 25 °C
DMSO	$(CH_3)_2SO$	78.14	-1.35	0.026	soluble
ethanol	CH_3CH_2OH	46.07	-0.31	2.8	1×10^6 mg/L
acetonitrile	CH_3CN	41.05	-0.34	not defined	>0.8 mg/L
n-dodecane	$CH_3(CH_2)_{10}CH_3$	170.33	6.98[c]	not applicable	0.005 mg/L [c]
n-heptane	$CH_3(CH_2)_5CH_3$	100.20	4.50[c]	3.9	2.4 mg/L [c]
1-hexene	$CH_2CH(CH_2)_3CH_3$	84.16	3.39	not defined	50 mg/L
n-hexane	$CH_3(CH_2)_4CH_3$	86.18	4.00[c]	8.3	10 mg/L [c]

[a] The data were from the websites; https://pubchem.ncbi.nlm.nih.gov, https://www.dispersetech.com/blog/post/properties-of-common-solvents.html, and http://www.stenutz.eu/chem/evaporation.php?s=1&p=2.

[b] Relative evaporation rates are obtained in a comparison of butyl acetate with vaporization rate as 1.0. Relative evaporation rate of higher than 3 indicates fast evaporation. Relative evaporation rate of lower than 0.8 indicates slow evaporation. Relative evaporation rate of water is 0.36.

[c] The data are from literature reference[4].

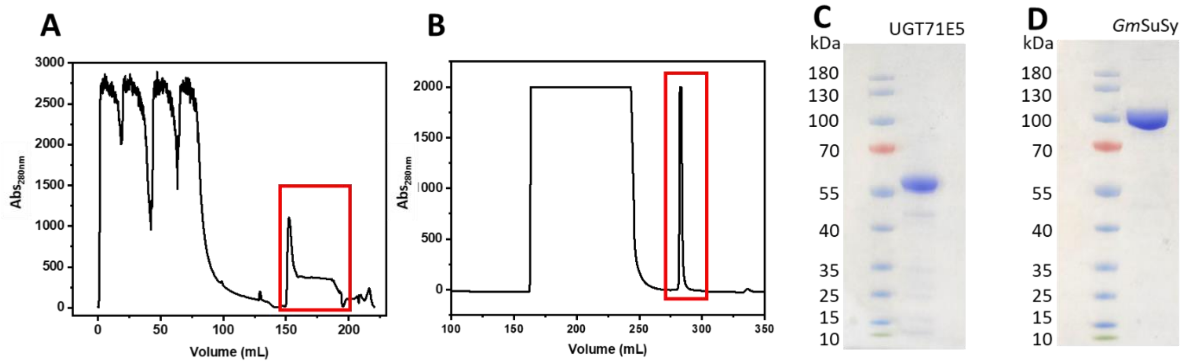

Supporting Figure S1. ÄKTA chromatograms and SDS-PAGE analysis from the purifications of UGT71E5 and *Gm*SuSy. (A-B) ÄKTA chromatogram from His-tag purification of UGT71E5 (A) and Strep-tag purification of *Gm*SuSy (B). UGT71E5 and *Gm*SuSy were eluted from the fractions highlighted in red. (C-D) SDS polyacrylamide gels showing purified UGT71E5 (C; 55.2 kDa) and *Gm*SuSy (D; 94.1 kDa). The purity was >95%.

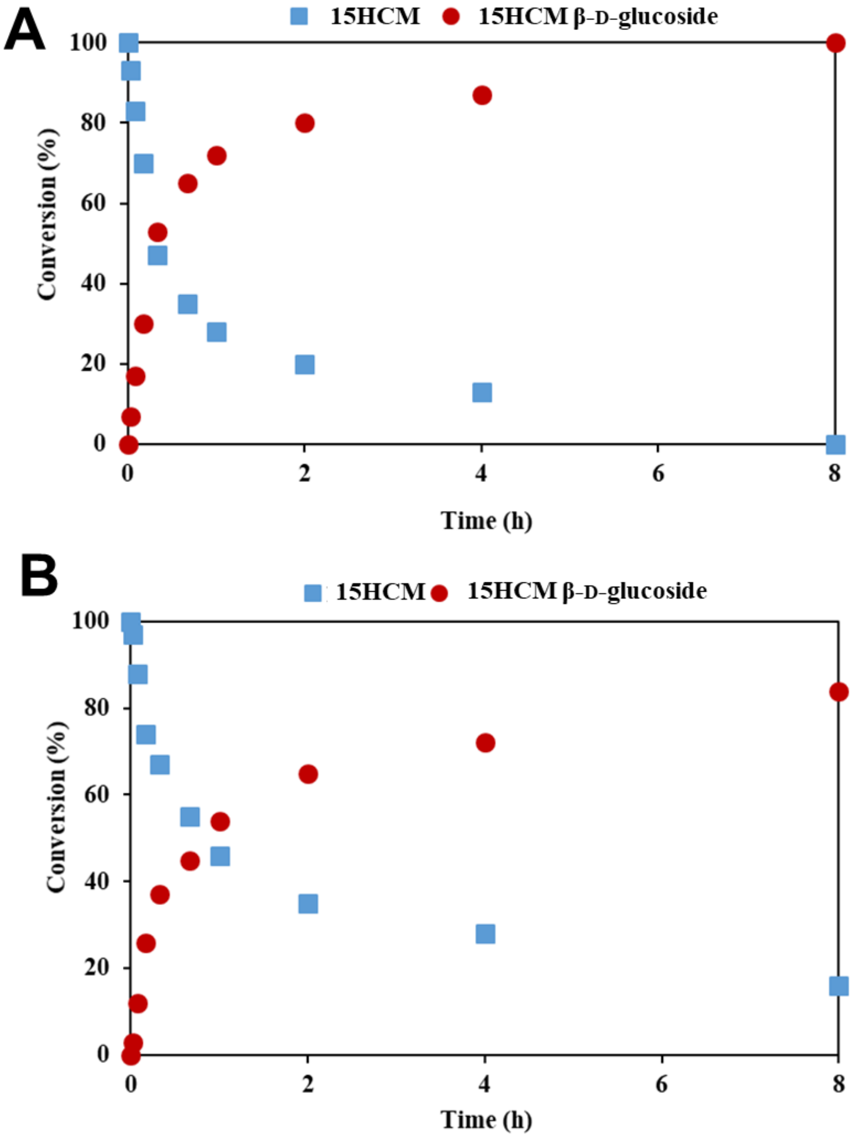

Supporting Figure S2. Time course of 15HCM conversion and 15HCM β-D-glucoside formation for measuring the activity of UGT71E5 with free (A) and HPβCD-encapsulated (B) 15HCM. Reactions (0.3 mL) contained 1.0 mM 15HCM (in 4% DMSO or HPβCD complexes, blue squares), 2.0 mM UDP-glucose, 5 mM $MgCl_2$ and 83.3 ng/ml UGT71E5 in Tris buffer (50 mM, pH 7.4), and were carried out at 30 °C with agitation rate of 500 rpm. Activities of UGT71E5 with free 15HCM (407 mU/mg) and 15HCM-HPβCD complex (320 mU/mg) were calculated based on initial 15HCM β-D-glucoside (red circles) formation (0–5 min). Both time courses in A and B show the average of two independent experiments N = 2, the standard deviation is ±6%.

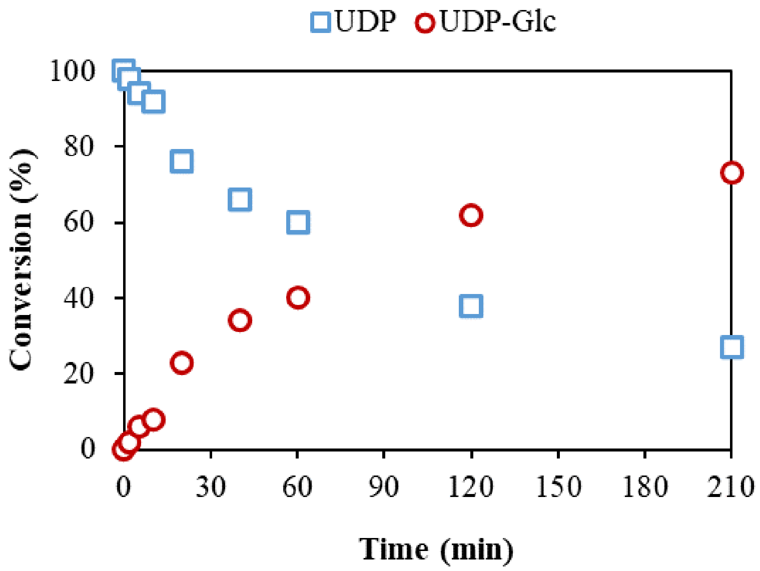

Supporting Figure S3. Time course of UDP conversion and UDP-glucose formation for measuring the activity of *Gm*SuSy. Reactions (0.3 mL) contained 500 mM sucrose, 2.0 mM UDP, 5 mM $MgCl_2$, 7.0 ng/mL *Gm*SuSy in Tris buffer (50 mM, pH 7.4), and were carried out at 30 °C with agitation rate of 500 rpm. Activity of *Gm*SuSy with UDP (3.63 U/mg) was calculated based on initial UDP-glucose formation (0–5 min). The time course shows the average of two independent experiments N = 2, the standard deviation is ±9%.

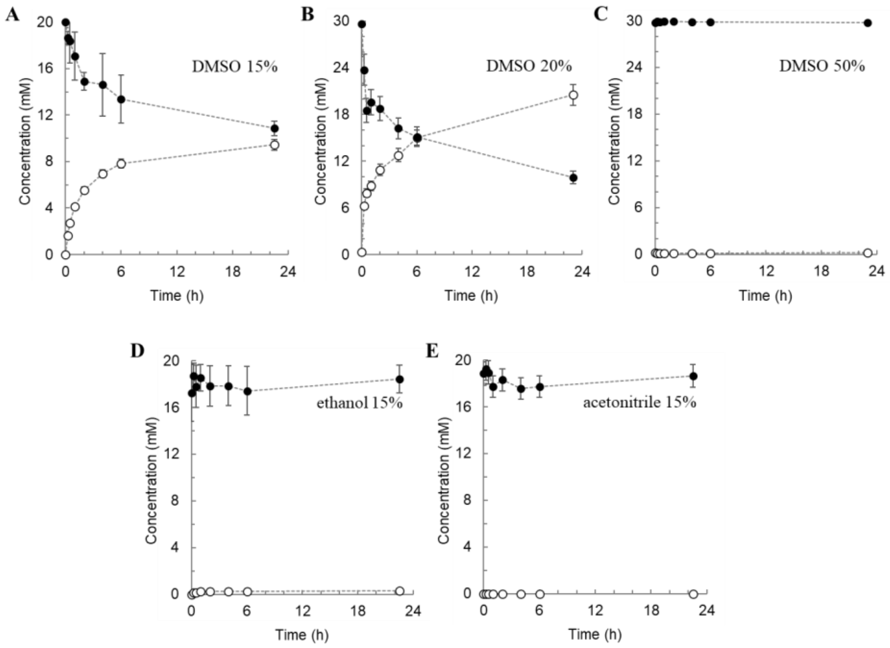

Supporting Figure S4. Glycosylation of 15HCM by UGT71E5 with water miscible organic solvents. DMSO (A, 15%; B, 20%; C, 50%), ethanol (D, 15%), and acetonitrile (E, 15%) were used. 15HCM β-D-glucoside (open circles) formed for 23 h was 9.4 mM (A, 15% DMSO) and 20.5 mM (B, 20% DMSO). The enzymatic conversion of 15HCM (closed circles) was hardly observed in the presence of 50% DMSO (C, ≤ 0.2 mM), 15% ethanol (D, ≤ 0.3 mM), and 15% acetonitrile (E, ≤ 0.05 mM). The data are summarized in Supporting Table S1. The standard deviations shown are from n ≥ 2 analytical determinations. Results shown are from a single time-course experiment N = 1. Monophasic reactions generally show high reproducibility with standard deviation estimated from repeated experiments at 10% DMSO (N ≥ 4) as ±6% or smaller.

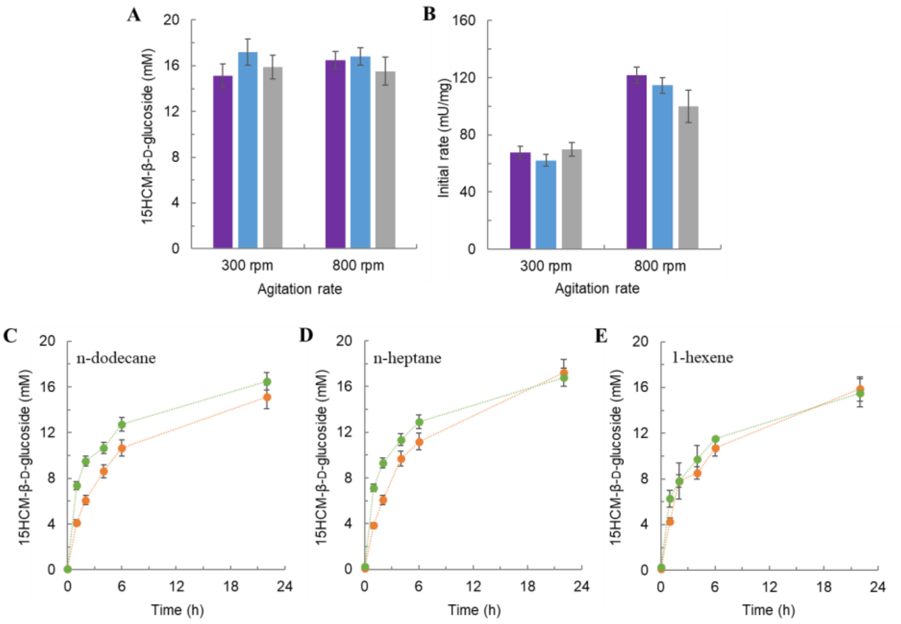

Supporting Figure S5. Acceleration of substrate transfer in biphasic reaction using varied agitation rates. The enzyme solution (0.4 mL; UGT71E5, 1 mg/mL) with UDP-glucose (40 mM) was used. 15HCM (100 mM) dissolved in n-dodecane, n-heptane, and 1-hexene (0.1 mL; 20% in total volume) was used. (A-B) Formed 15HCM β-D-glucosides after incubation for 22 h (A) and initial rates of glycosylation of 15HCM (B). The reactions with n-dodecane (purple bars), n-heptane (blue bars), and 1-hexene (light gray bars) showed increased initial rates of 15HCM glycosylation at higher agitation rates. Formed β-D-glucoside of 15HCM however, did not increase by higher agitation rates. (C-E). Time-dependent formation of 15HCM β-D-glucoside with n-dodecane (C), n-heptane (D), and 1-hexene (E). Formation of 15HCM β-D-glucoside was shown consistently at 300 rpm (orange circles) and 800 rpm (green circles). The standard deviations shown are from n ≥ 2 analytical determinations. Results shown are from a single time-course experiment N = 1. Biphasic reactions generally show lower reproducibility than monophasic reactions. Standard deviation estimated from repeated experiments with dodecane (N ≥ 4) as ±10% or smaller.

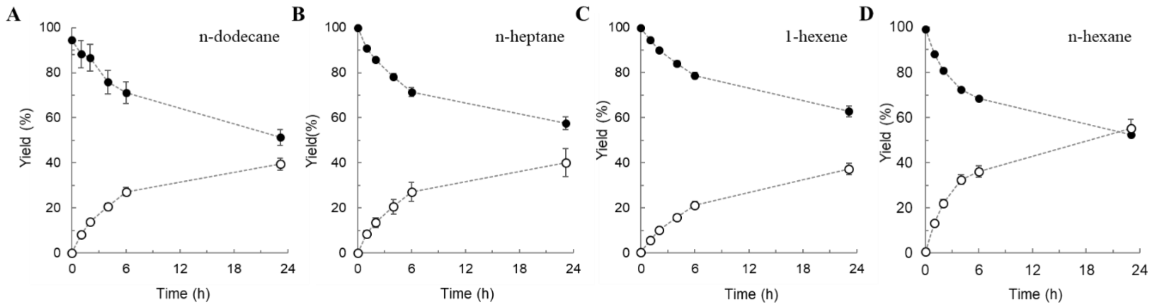

Supporting Figure S6. Glycosylation of 15HCM in 1:1 organic-aqueous biphasic system. n-dodecane (A), n-heptane (B), 1-hexene (C), and n-hexane (D) were used as organic phase (0.3 mL) containing 15HCM (30 mM). The enzyme solution of UGT71E5 (0.3 mL, 1.5 mg/mL) with UDP-glucose (60 mM) was used as aqueous phase. 15HCM (closed circles) was observed in both organic and aqueous phases. The β-D-glucoside form of 15HCM (open circles) was observed in aqueous phase (A, B) or in both phases (C, D). The partition of 15HCM and its β-D-glucoside under the condition used is shown in Supporting Figure S7. The data were summarized in Table 1. The standard deviations shown are from $n \geq 2$ analytical determinations. Results shown are from a single time-course experiment $N = 1$. Biphasic reactions generally show lower reproducibility than monophasic reactions. Standard deviation estimated from repeated experiments with dodecane ($N \geq 4$) as ±10% or smaller.

1:1 organic-aqueous biphasic reaction

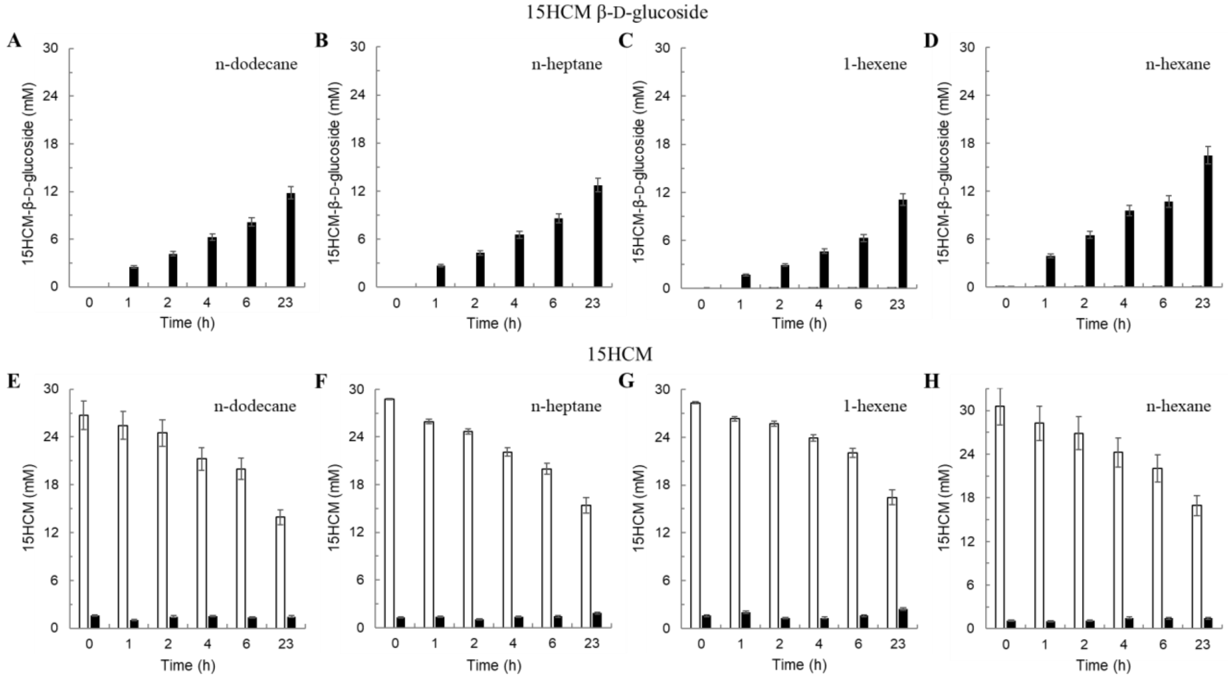

Supporting Figure S7. Partition of 15HCM β-D-glucoside and 15HCM in 1:1 organic-aqueous biphasic reaction.15HCM β-D-glucoside (A-D) was mainly observed in aqueous phase (black bars), while 15HCM (E-H) was largely detected in organic phase (white bars). The β-D-glucoside was not detectable in n-dodecane (A) and n-heptane (B). Small amounts of 15HCM β-D-glucoside were detected in 1-hexene (C, white bars, 0.10 ± 0.02 mM; N ≥ 3) and n-hexane (D, white bars, 0.11 ± 0.03 mM; N ≥ 3) due to relatively high-water solubility (Supporting Table S2). 15HCM in aqueous phase (E-H, black bars) was 1.4 ± 0.2 mM (E, n-dodecane; N ≥ 3), 1.4 ± 0.4 mM (F, n-heptane; N ≥ 3), 1.7 ± 0.5 mM (G, 1-hexene; N ≥ 3), and 1.4 ± 0.2 mM (H, n-hexane; N ≥ 3). The data are also shown and summarized in Supporting Figure S6 and Table 1. The standard deviations shown are from n ≥ 2 analytical determinations. Results shown are from a single time-course experiment N = 1. Biphasic reactions generally show lower reproducibility than monophasic reactions. Standard deviation estimated from repeated experiments with dodecane (N ≥ 4) as ±10% or smaller.

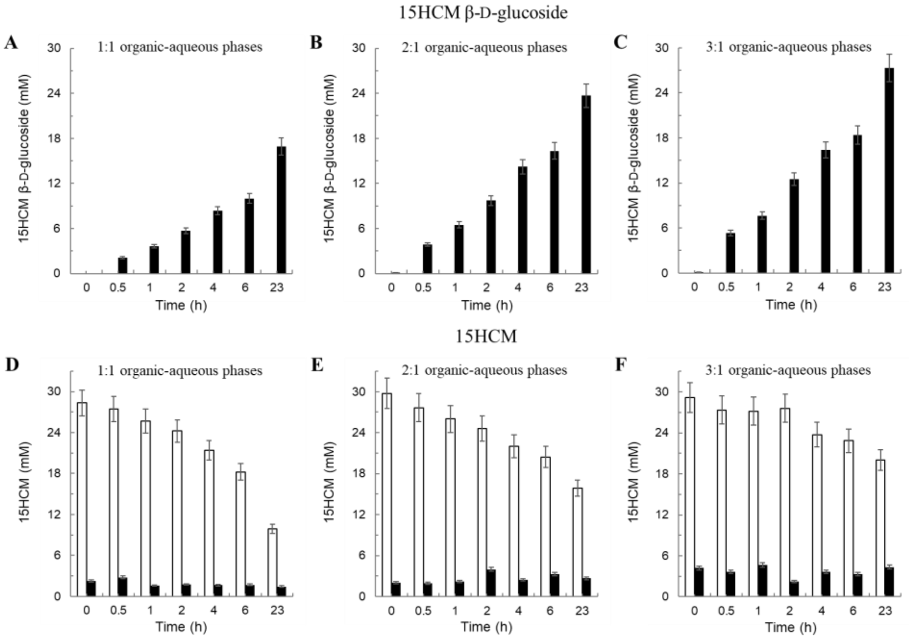

Supporting Figure S8. Partition of 15HCM β-D-glucoside and 15HCM in organic-aqueous biphasic reaction with n-dodecane and 10% DMSO. Formation of 15HCM β-D-glucoside (A-C) and consumption of 15HCM (D-F) are shown by time. Volumetric ratios of n-dodecane to aqueous solution were 1:1 (A, D), 2:1 (B, E), and 3:1 (C, F). 15HCM β-D-glucoside was observed solely in aqueous phase (black bars). 15HCM was mainly found in organic phase (white bars). 15HCM in aqueous phase (black bars) with 10% DMSO was 1.9 ± 0.5 mM (N ≥ 3) (D), 2.6 ± 0.7 mM (N ≥ 3) (E), and 3.7 ± 0.8 mM (N ≥ 3) (F). The data were also shown and summarized in Figure 4 and Table 1. The standard deviations shown are from n ≥ 2 analytical determinations. Results shown are from a single time-course experiment N = 1. Biphasic reactions generally show lower reproducibility than monophasic reactions. Standard deviation estimated from repeated experiments with dodecane (N ≥ 4) as ±10% or smaller.

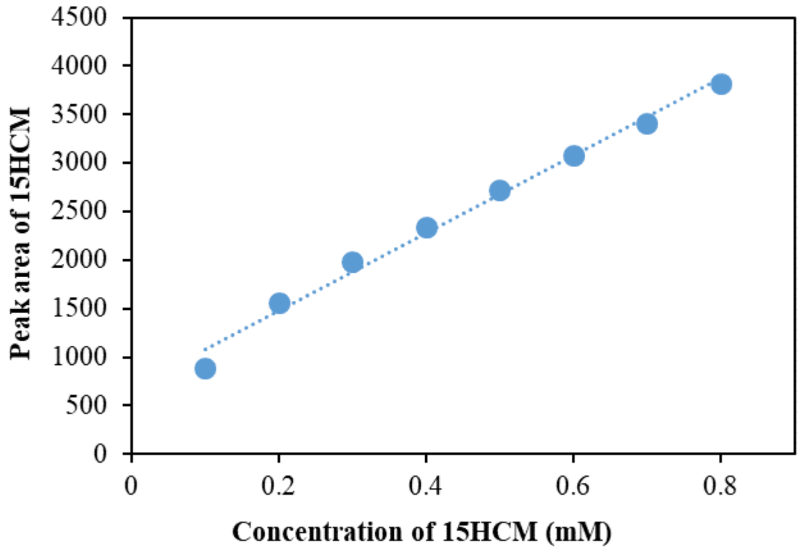

Supporting Figure S9. Calibration line of 15HCM. The relationship between the concentration of 15HCM and corresponding HPLC integrated peak areas (at 203 nm, blue circles) for the measurement of the actual concentration of 15HCM-HPβCD inclusion complexes is shown.

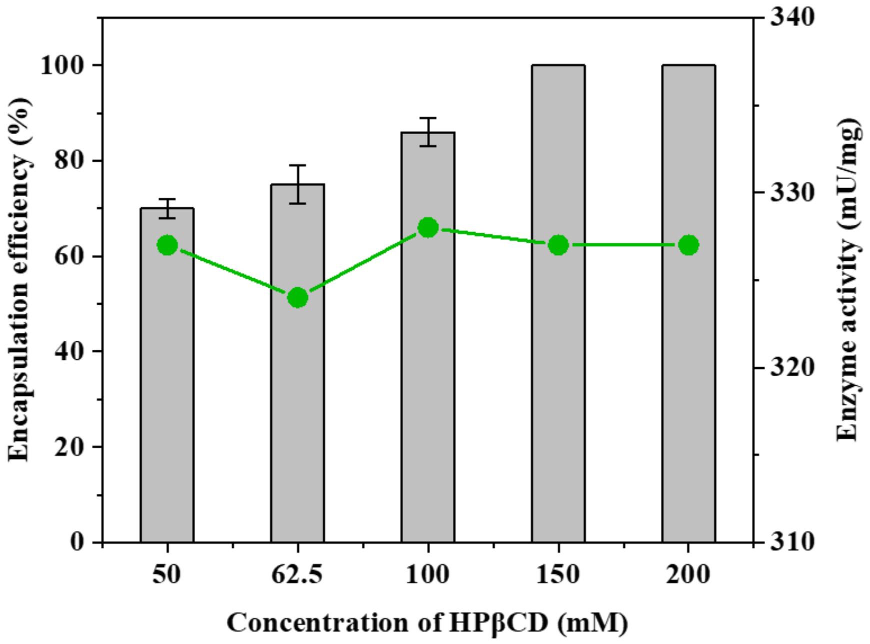

Supporting Figure S10. Encapsulation efficiency (gray bars) and UGT71E5 activity (green circles) with different concentrations of HPβCD. 15HCM (50 mM) was added with varied concentration of HPβCD (50–200 mM) to form the inclusion complex. Reactions (0.3 mL) of enzyme activity assay contained 1.0 mM 15HCM-HPβCD, 2.0 mM UDP-glucose, 5 mM $MgCl_2$, 0.12 mg/mL UGT71E5 in Tris buffer (50 mM, pH 7.4), and were carried out at 30 °C with agitation rate of 500 rpm. Standard deviations are from N = 2 experiments.

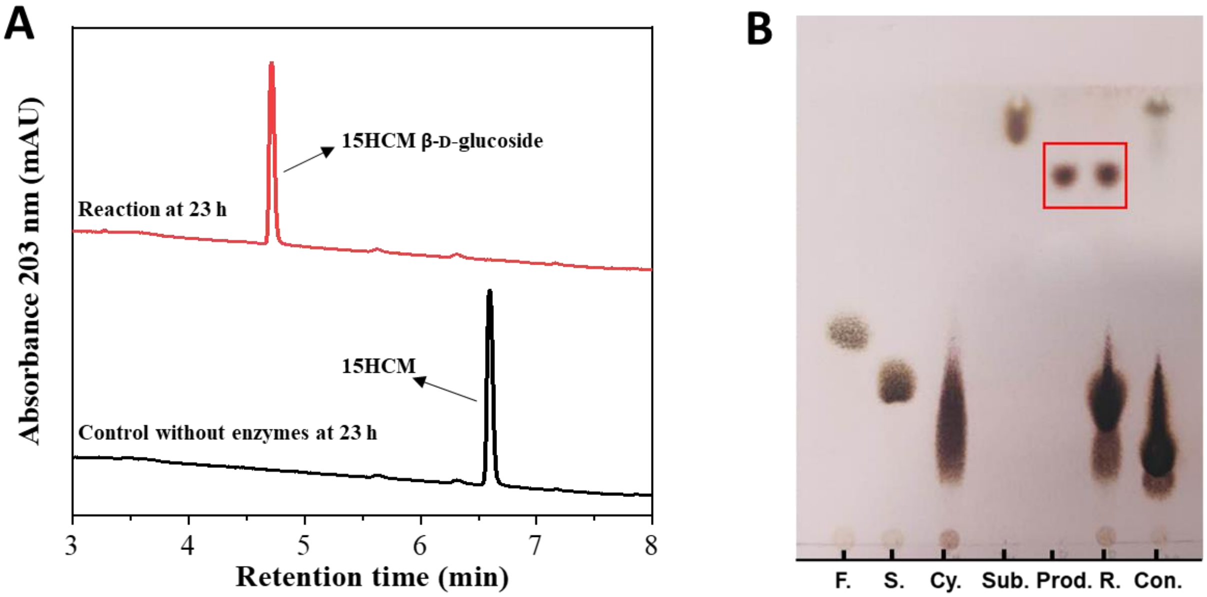

Supporting Figure S11. (A) Reverse phase HPLC chromatograms of the reaction mixture from the synthesis of 15HCM β-D-glucoside (red; full conversion to 15HCM β-D-glucoside) and control reaction without enzymes (black) at 23 h time points. (B) TLC analysis of the reaction mixtures at 23 h from the same samples as in (A). F. = fructose, S. = sucrose, Cy. = HPβCD, Sub. = 15HCM, Prod. = 15HCM β-D-glucoside, R. = reaction mixture, Con. = control reaction without enzymes. Note: Sucrose and HPβCD from the control reaction run slower on the silica plate when compared to the enzymatic reaction. This phenomenon was observed in each control reaction containing 50 mM 15HCM (N = 3).

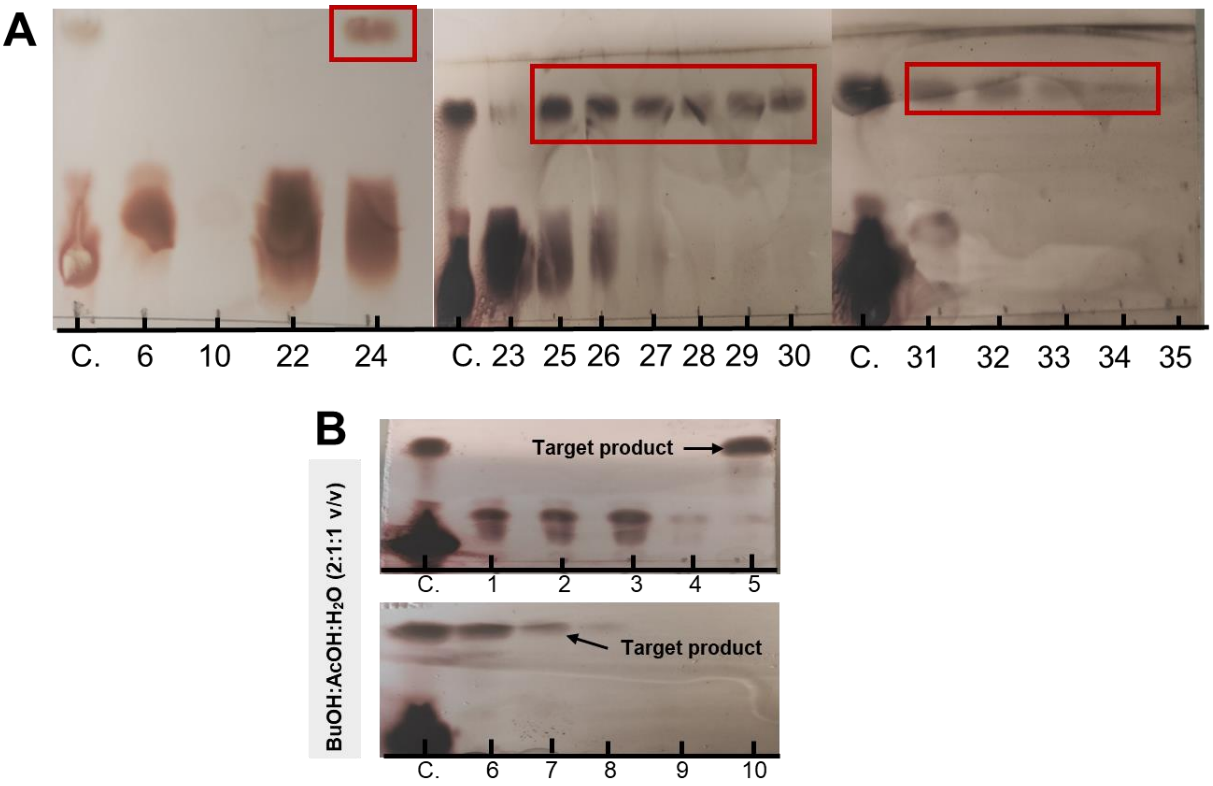

Supporting Figure S12. TLC plates from the isolation of 15HCM β-D-glucoside by two-step silica column chromatography. (A) TLC analysis of the fractions eluted from the C18 column. The target product is highlighted with a red frame. (B) TLC analysis of the fractions eluted from the silica 60 column. C. = reaction mixture prior to purification.

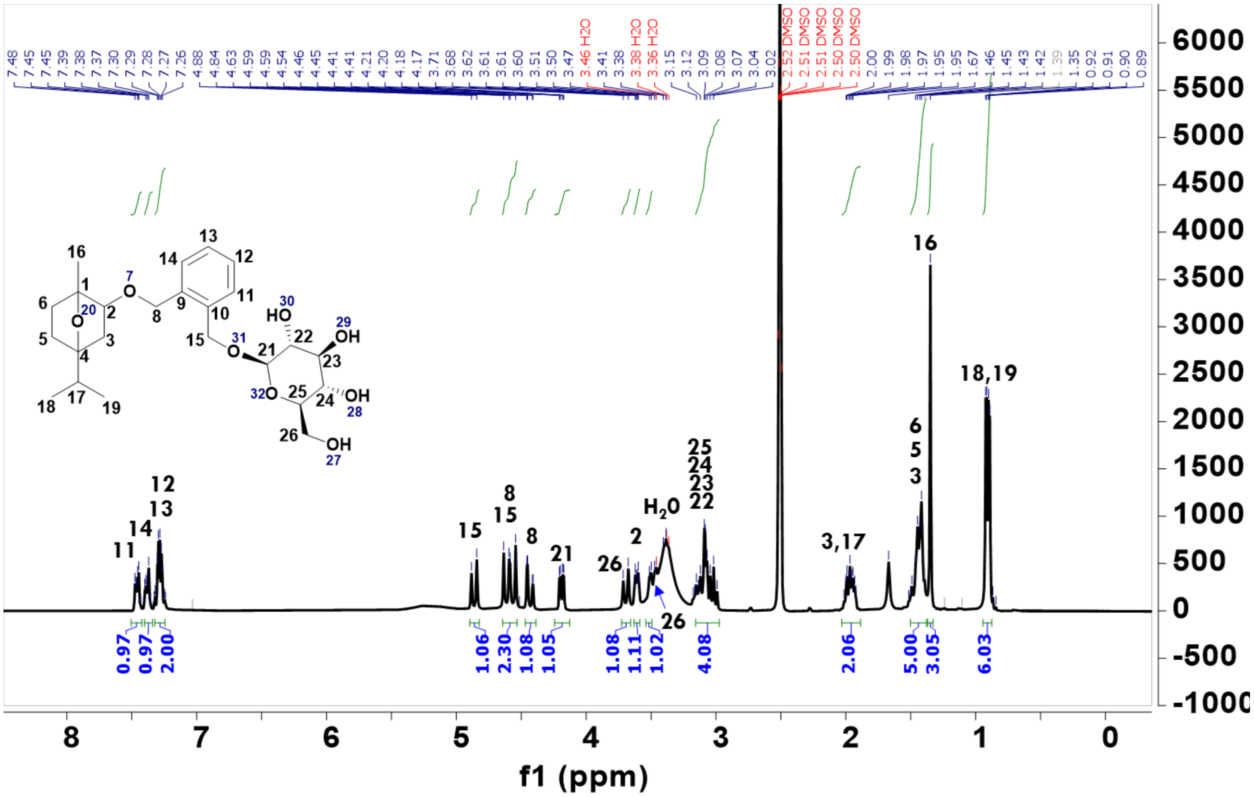

Supporting Figure S13. ^{1}H NMR spectrum (300 MHz, DMSO-d_6) of 15HCM β-D-glucoside. δ: 7.51 – 7.42 (m, 1H), 7.38 (m,1H), 7.29 (m, 2H), 4.86 (d, J = 12.3 Hz, 1H), 4.64 – 4.53 (m, 2H), 4.43 (dd, J = 12.7, 1.7 Hz, 1H), 4.19 (dd, J = 7.7, 2.6 Hz, 1H), 3.70 (d, J = 11.6 Hz, 1H), 3.61 (m, 1H), 3.51 (d, J = 4.2 Hz, 1H), 3.16 – 2.97 (m, 4H), 1.97 (m, 2H), 1.44 (m, 5H), 1.35 (s, 3H), 0.91 (dd, J = 6.8, 2.8 Hz, 6H).

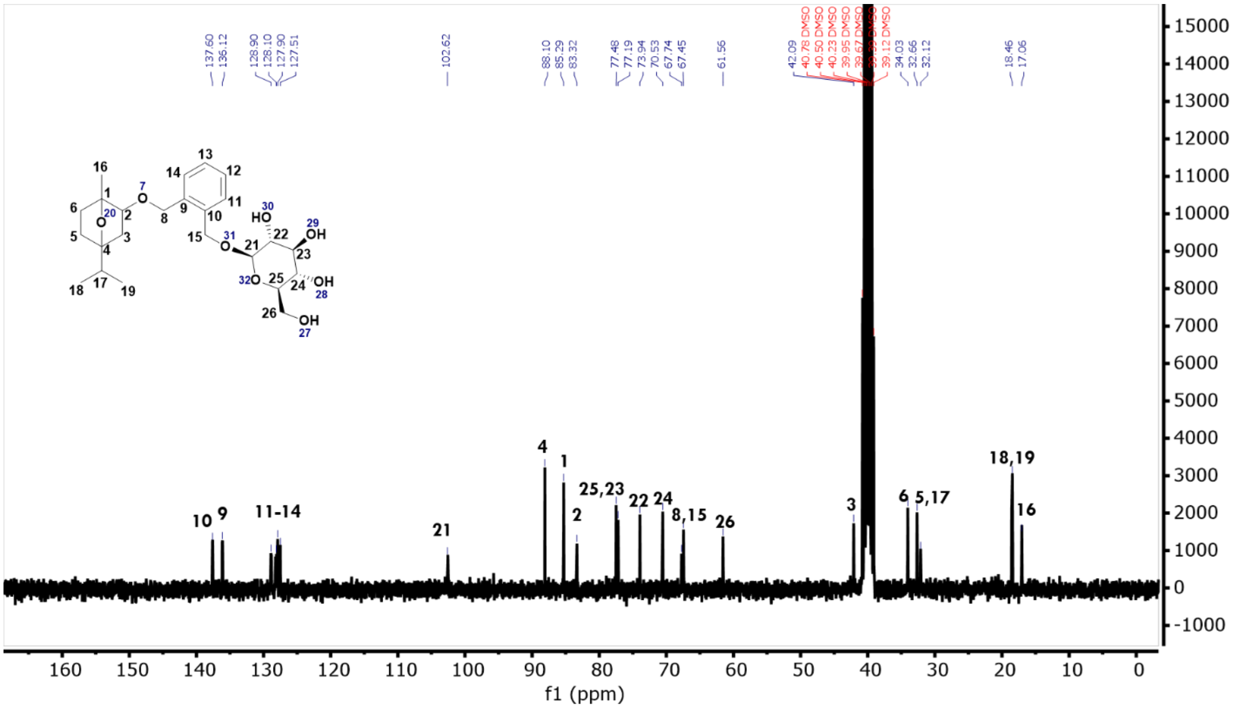

Supporting Figure S14. ^{13}C NMR spectrum (75 MHz, DMSO-d_6) of 15HCM β-D-glucoside. δ: 137.60, 136.12, 128.90, 128.10, 127.90, 127.51, 102.62, 88.10, 85.29, 83.32, 77.48, 77.19, 73.94, 70.53, 67.74, 67.45, 61.56, 42.09, 34.03, 32.66, 32.12, 18.46, 17.06.

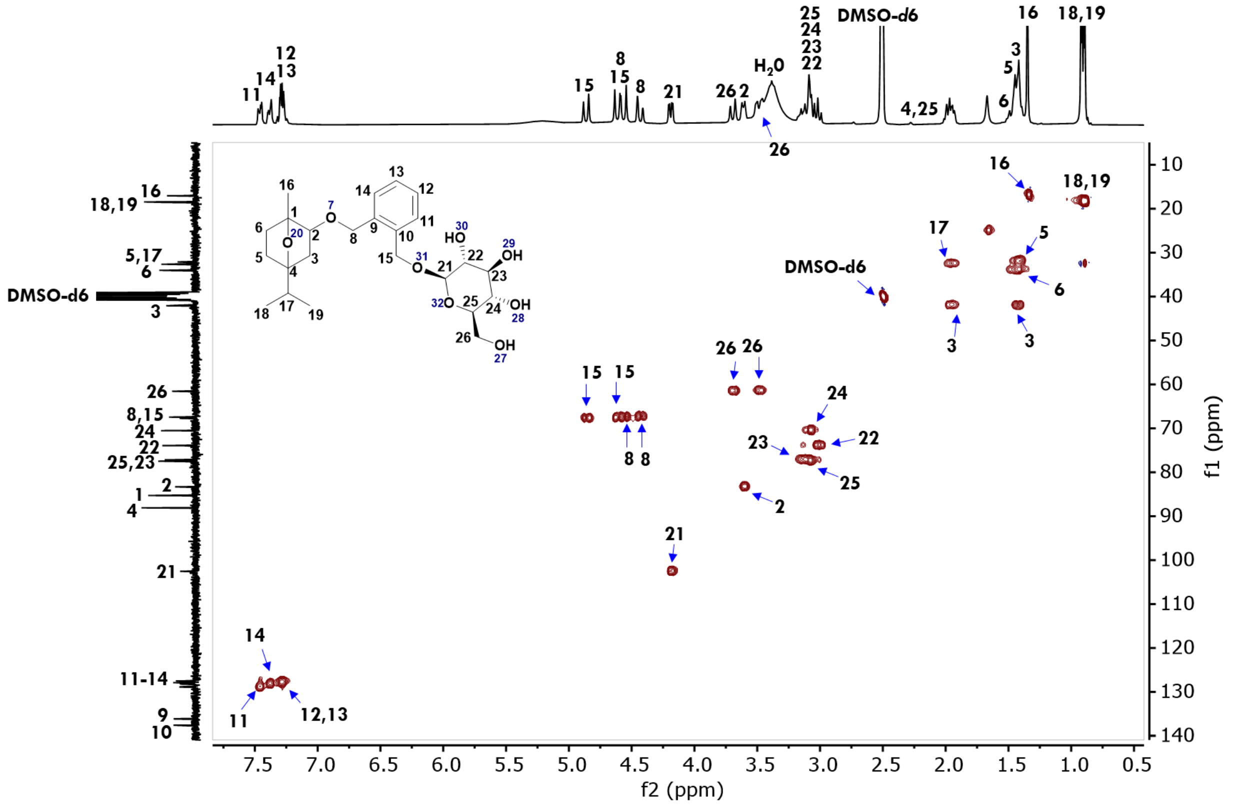

Supporting Figure S15. Heteronuclear single quantum coherence spectroscopy (HSQC) analysis of 15HCM β-D-glucoside (300 MHz, DMSO-d_6).

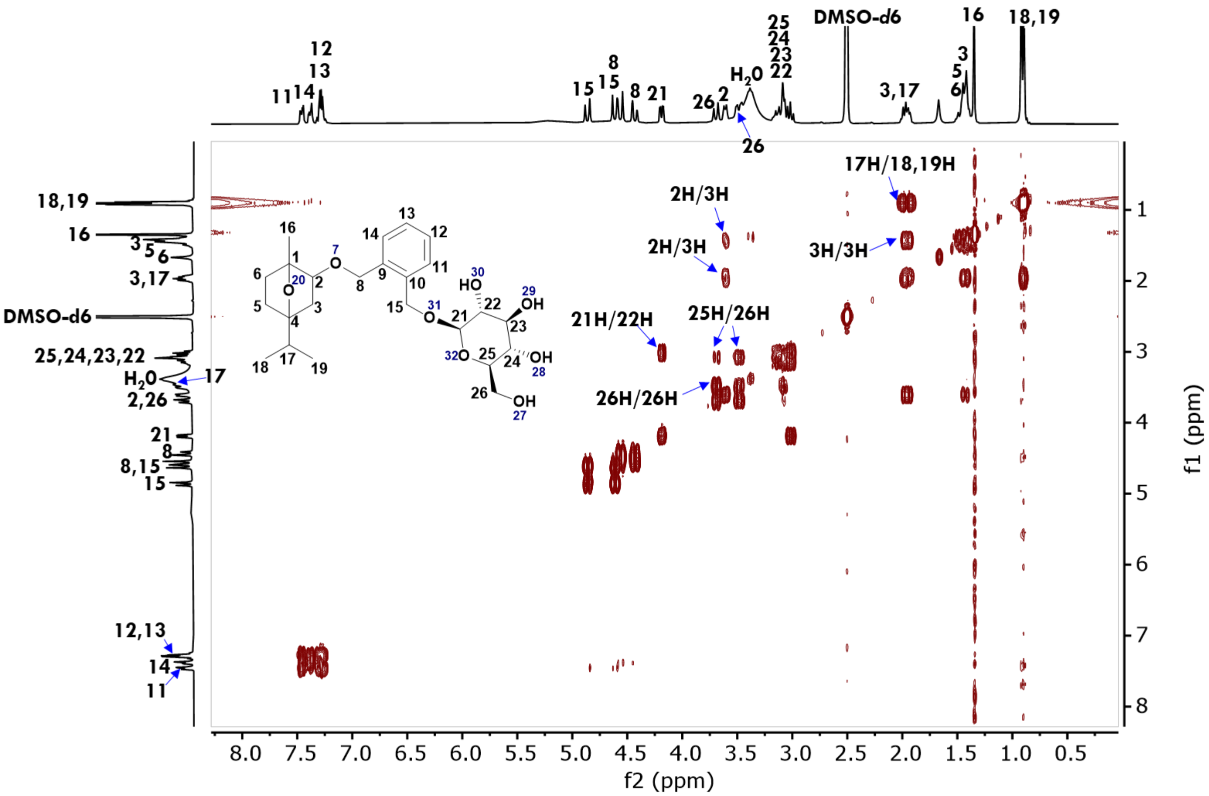

Supporting Figure S16. Correlated spectroscopy (COSY) analysis of 15HCM β-D-glucoside (300 MHz, DMSO-d_6).

References

(1) Xie, K.; Chen, R.; Chen, D.; Li, J.; Wang, R.; Yang, L.; Dai, J. Enzymatic *N*-glycosylation of diverse arylamine aglycones by a promiscuous glycosyltransferase from *Carthamus tinctorius*. *Adv. Synth. Catal.* **2017**, *359*, 603–608.

(2) Jung, J.; Schmölzer, K.; Schachtschabel, D.; Speitling, M.; Nidetzky, B. Selective β-mono-glycosylation of C15 hydroxylated metabolite of the agricultural herbicide cinmethylin using leloir glycosyltransferases. *J. Agric. Food Chem.* **2021**, *69*, 5491–5499.

(3) Bungaruang, L.; Gutmann, A.; Nidetzky, B. Leloir glycosyltransferases and natural product glycosylation: biocatalytic synthesis of the *C*-glucoside nothofagin, a major antioxidant of redbush herbal tea. *Adv. Synth. Catal.* **2013**, *355*, 2757–2763.

(4) Rosinha Grundtvig, I. P.; Heintz, S.; Krühne, U.; Gernaey, K. V.; Adlercreutz, P.; Hayler, J. D.; Wells, A. S.; Woodley, J. M. Screening of organic solvents for bioprocesses using aqueous-organic two-phase systems. *Biotechnol. Adv.* **2018**, *36*, 1801–1814.
